# Catalytic Zinc Complexes for Phosphate Diester Hydrolysis**

**DOI:** 10.1002/anie.201400335

**Published:** 2014-06-11

**Authors:** Emmanuel Y Tirel, Zoë Bellamy, Harry Adams, Vincent Lebrun, Fernanda Duarte, Nicholas H Williams

**Affiliations:** Department of Chemistry, Sheffield UniversitySheffield (UK); Department of Cell and Molecular Biology, Uppsala UniversityUppsala (Sweden)

**Keywords:** bioinorganic chemistry, DNA cleavage, enzyme models, kinetics, zinc

## Abstract

Creating efficient artificial catalysts that can compete with biocatalysis has been an enduring challenge which has yet to be met. Reported herein is the synthesis and characterization of a series of zinc complexes designed to catalyze the hydrolysis of phosphate diesters. By introducing a hydrated aldehyde into the ligand we achieve turnover for DNA-like substrates which, combined with ligand methylation, increases reactivity by two orders of magnitude. In contrast to current orthodoxy and mechanistic explanations, we propose a mechanism where the nucleophile is not coordinated to the metal ion, but involves a tautomer with a more effective Lewis acid and more reactive nucleophile. This data suggests a new strategy for creating more efficient metal ion based catalysts, and highlights a possible mode of action for metalloenzymes.

Substantial efforts have been made to create metal ion complexes that are effective catalysts for phosphate ester hydrolysis.[1] These compounds provide insight into how biological catalysts might function, and hold the promise of creating novel therapeutics or laboratory agents for manipulating nucleic acids.[2] The challenges of sufficient activity to function usefully under biological conditions and achieving turnover remain. Herein we report how incorporating a hydrated aldehyde as a nucleophile can enhance reactivity and lead to turnover. Our mechanistic explanation provides a new strategy for designing metal ion complexes with nuclease activity.

In developing artificial metal ion complexes to cleave RNA, the 2′OH group provides an intramolecular nucleophile which can be exploited.[3] For DNA, this is not possible, and the most effective strategies to date have used metal-ion-coordinated nucleophiles to enhance the attack at phosphorus. Chin and co-workers established that the effectiveness of this nucleophile can depend strongly on ligand structure.[4] If this nucleophile is part of the ligand structure, then its efficiency can be enhanced through careful design, and substantial rate enhancements achieved compared to that a metal-bound hydroxide. However, the flaw in this strategy is that the product is a phosphorylated ligand which is very stable, and so the complexes are not catalytic.

A potential solution to this problem is suggested by the hydrolysis of model compounds also containing keto or aldehyde groups.[5] Bender and Silver showed that benzoate ester hydrolysis can be accelerated 10^5^-fold by the presence of an *ortho* aldehyde group. This hydrate form of the aldehyde provides an effective nucleophile, thus producing a product which can readily decompose to reform the carbonyl.[6] Similar effects have been reported for phosphate ester cleavage.[7] To create a catalytic system, Menger and Whitesell incorporated aldehydes into micellar head groups, and these aggregates showed both enhanced activity and turnover.[8] Interestingly, recent work with sulfatases and phosphonohydrolases has shown that a formyl glycine residue in the active site is believed to act as a nucleophile through its hydrated form. It has been speculated that this nucleophile may facilitate the broad substrate tolerance of these enzymes as the covalently modified enzyme can decompose through a common mechanism (reforming the aldehyde by eliminating the derivatized hydroxy) which is independent of the functional group being hydrolyzed.[9]

Our designs are based on pyridyl zinc complexes with a simple alcohol chain as a nucleophile (**1**; Scheme 1). The propylene linker is much more reactive than the ethylene analogue, or complexes which do not have an alkoxy nucleophile. It has been shown that 2-amino substituents on the pyridyl ring can have a large effect on reactivity, and is presumed to be due to potential hydrogen bonding with the substrate.[10] We decided not to incorporate an amino group in this work so as to avoid condensation reactions with the aldehyde. Instead, we incorporated methyl groups into the 2-position of the pyridyl ring (**2**), thus reflecting the steric demands on the 2-amino group albeit with a minimal capacity to provide hydrogen-bond donors. Modifying the substrate binding pocket this way has also been suggested to provide a hydrophobic cavity which could enhance electrostatic interactions.[11] We were not able to oxidize the alcohol in **2**. This reaction always led to loss of the side chain, presumably because of elimination reactions involving the central methylene group, and so we synthesized **4** by oxidizing **3**. The reaction we have studied is the cleavage of bis(*p*-nitrophenyl) phosphate (BNPP) as a convenient model for DNA cleavage under completely aqueous conditions at 25 °C, thus allowing comparison of our data with that of previous reports.

**Scheme 1:**
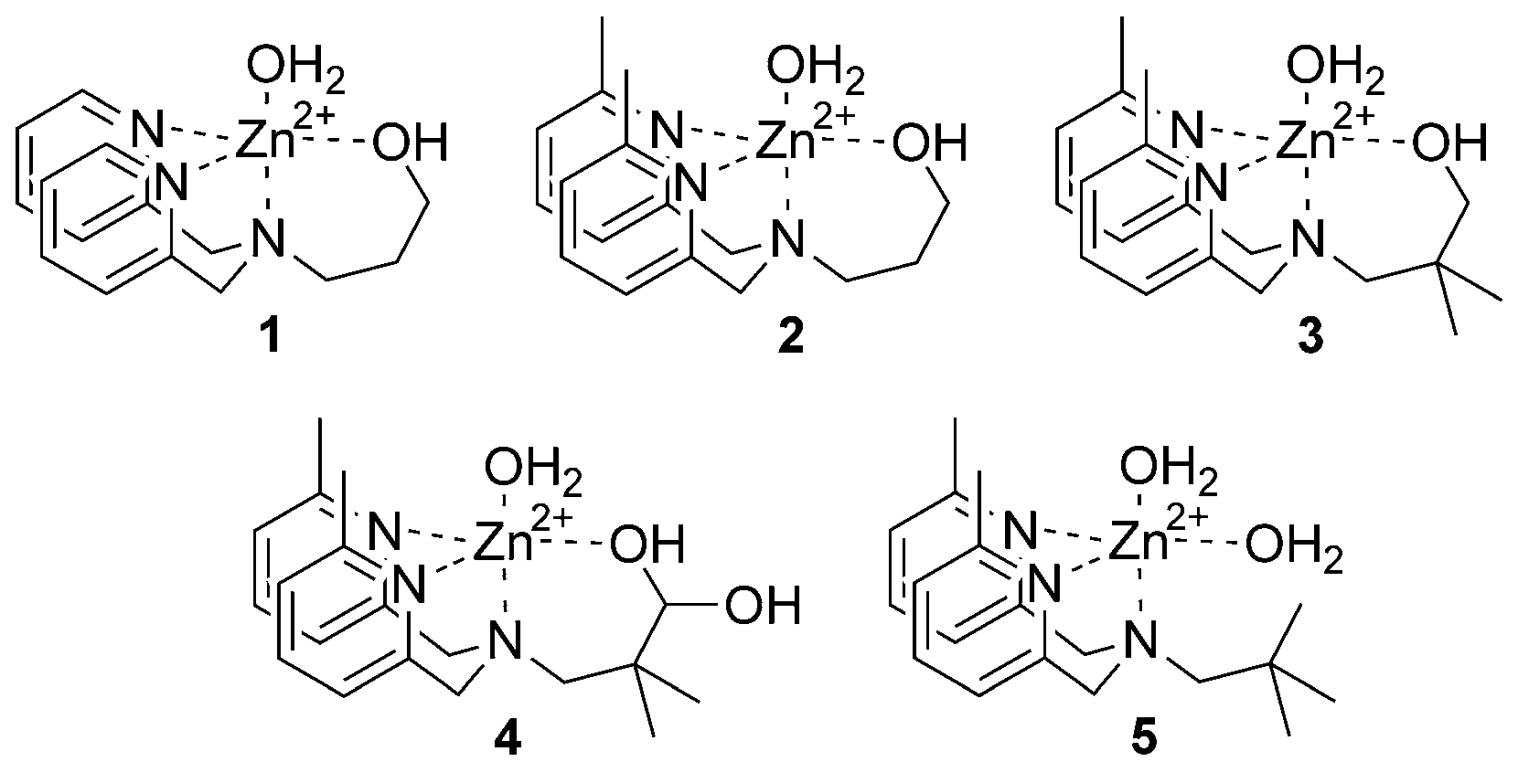
Zinc complexes used in this study.

The cleavage reaction shows a first-order dependence on increasing complex concentration (0.2–1 mm) for **2**, and the pH dependence reveals a bell-shaped pH rate profile (Figure 1). ^31^P NMR spectroscopy confirmed that **2** is phosphorylated to produce a stable diester (no further reaction over 20 days), and when excess substrate is used, only one equivalent reacts with the complex. We note that the introduction of a methyl group into the 2-position of the pyridyl ligand provides a rate acceleration of about fivefold in the maximum rate compared to the unsubstituted complex **1** (Table 1). This acceleration suggests that a steric effect between substrate and ligand provides a moderate improvement in efficiency, and that it is likely to be a partial factor in the 230-fold rate increase induced by using 2-amino substitutions on the pyridyl ligands.

**Figure 1:**
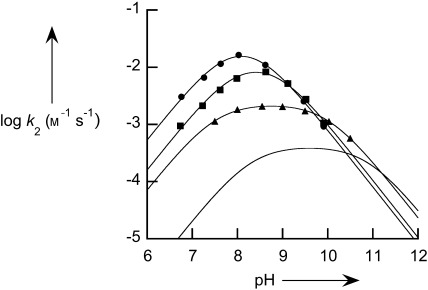
pH rate profile for the cleavage of BNPP catalyzed by 1 (no symbols; from reference [12]), by 2 (triangles), by 3 (squares), and by 4 (circles) at 25 °C ([buffer]=0.05 m). Solid lines are from fitting Equation (1) to the data.

**Table 1:** Collected kinetic parameters from pH rate profiles.

Compound	p*K*_a_^1^	p*K*_a_^2^	*k*_2_^max^ [m^−1^ s^−1^]
**1**^[a]^ (H_2_O)	8.3^[a]^	10.9^[a]^	4.2×10^−4[a]^
**2** (H_2_O)	7.50±0.03	10.00±0.02	2.3±0.04×10^−3^
**2** (MeOH)	8.2±0.2	10.3±0.1	4.7±0.6
**3** (H_2_O)	7.9±0.1	8.9±0.1	1.3±0.2×10^−2^
**3** (MeOH)	9.5±0.1^[b]^	10.1±0.1^[b]^	23±5
**4** (H_2_O)	7.7±0.1	8.4±0.1	2.9±0.3×10^−2^
**4′** (MeOH)	8.6±0.1	9.7±0.1	9±1×10^−2^

[a] Reference [12]. [b] The p*K*_a_ values are constrained to differ by 0.6 units, the closest that they can be without cooperative deprotonation being required.[13]

Similar first-order behavior is observed when **4** reacts with BNPP at high pH, but at lower pH a nonlinear dependence on concentration is apparent. We analyze this in terms of relatively weak binding between the ligand and Zn^II^, as confirmed by a potentiometric titration (see the Supporting Information). At low pH, ligand protonation competes with Zn complexation and the decrease in activity at low concentrations is due to the dissociation of Zn from the ligand. Adding additional Zn ions increases the rate of the reaction, thus showing a saturation curve with an apparent binding constant which matches the parameters derived from the titration data (see the Supporting Information), and leads to a linear dependence on complex concentration. Plotting the limiting second-rate constants for the reaction catalyzed by **4** at different pH values reveals a bell-shaped pH rate profile, and the maximal activity of **4** is 70-fold greater than that for **1**, and 13-fold greater than that for **2**. The bell-shape pH rate profiles are fit to a reaction scheme where the singly deprotonated species [Scheme 2 and Eq. (1)] is the kinetically active ionic species.

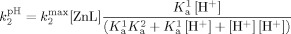
(1)

**Scheme 2:**
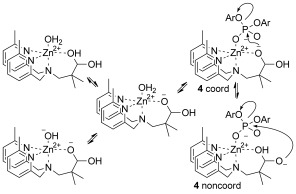
Mechanistic scheme for reaction of 4 with BNPP.

In contrast to **2**, when the reaction is monitored by ^31^P NMR spectroscopy, the observed product is 4-nitrophenyl phosphate monoester, with no evidence of a transesterification intermediate, and the complex completely hydrolyzes 5 equivalents of substrate (no substrate observable after 50 days), thus demonstrating catalytic turnover. As this observation is also consistent with a metal-bound hydroxide acting as the nucleophile, rather than the ligand, we tested **5** for activity: this complex showed no detectable activity under the reaction conditions for over 46 days (see the Supporting Information), and so we infer that **4** reacts through the hydrated aldehyde nucleophile to form a transient intermediate which rapidly decomposes to form the original complex. When **4** is recrystallized in the presence of methanol, the hemiacetal **4′** is isolated and clearly shows that the zinc ion can be coordinated by the hemiacetal form of the aldehyde side chain, thus corroborating this interpretation (Figure 2).

**Figure 2:**
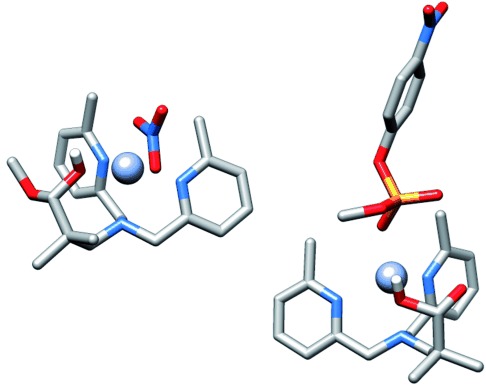
a) Representation of the X-ray crystal structure of 4′ isolated from methanol (hydrogen atoms and noncoordinated nitrate omitted for clarity, except for OH coordinated to Zn). b) Optimized structure of the monodeprotonated form of 4, with methyl 4-nitrophenyl phosphate bound, at the HF/6-31+G*/LANL2DZ level of theory, using SMD continuum solvent model (hydrogen atoms omitted for clarity, except for OH coordinated to Zn).

The compound **3** behaves essentially the same way as **2** (undergoing a single reaction to produce a stable product), but surprisingly is also significantly more reactive than **2** (sevenfold). There is no obvious explanation why methylating the side chain should lead to a more active complex: the Thorpe–Ingold effect usually enhances the formation of cyclic compounds, but it seems more likely that perturbation of the local solvation shell or indirect steric effects (e.g. with the pyridyl groups) may affect the zinc coordination site and its Lewis acidity.

As well as introducing turnover, the germinal diol nucleophile has a lowered p*K*_a_ value, thus bringing the maximum activity closer to physiological pH. However, Mancin and co-workers demonstrated that the cost of lowering the p*K*_a_ value of zinc-coordinated alkoxides is to reduce the activity of the nucleophile towards BNPP, and the overall effect is a less reactive complex at all pH values, albeit with a maximum closer to pH 7. Thus, the maximal reactivity of **4** is expected to be some 65-fold lower than for **3**, assuming that the geminal hydroxy group has a similar effect on the OH p*K*_a_ value as a geminal trifluoromethyl group, rather than twofold more reactive.[14]

This unexpected increase in activity leads us to question whether the active nucleophile is coordinated to the zinc ion (**4** coord; Scheme 2) as has been generally assumed for these type of metal ion complexes. In **4**, the uncoordinated OH is an alternative nucleophile, particularly if we consider the tautomer where it is deprotonated (**4** non-coord; Scheme 2) as the reactive species. For this to be a viable possibility, the *β*_nuc_ for the reactions needs to be significant so that the greater reactivity of the higher p*K*_a_ anion can compensate adequately for the unfavorable equilibrium between the tautomers. The data of Mancin and co-workers[14] suggest that this is plausible. An estimate of *β*_nuc_≈1 can be made from their data, and this predicts similar overall reactivity for both tautomers when nucleophilicity and concentration are combined. This value still predicts lower overall reactivity compared to that of the more basic alkoxy nucleophiles such as that in **3**. However, we note that if the zinc is coordinated by OH instead of O^−^, then presumably it will be a more effective Lewis acid to activate the BNPP, and combined with a different geometry for attack (involving formation of a six-membered instead of four-membered ring), this mechanism provides a plausible explanation as to why **4** has slightly higher maximal reactivity than **3**.

It is not immediately obvious from the X-ray structure (Figure 2) that the uncoordinated oxygen atom can act as a nucleophile[15] towards a substrate coordinated to the Zn ion, so we used computational methods to explore whether more promising geometries are readily accessible. Starting from the X-ray structure of **4′**, we converted the methoxy group into a hydroxy group, and the coordinated nitrate to a water molecule, then minimized the structure using the Hartree–Fock (HF) level of theory to optimize the geometry, and DFT calculations to carry out single-point energy calculations. The computational methodology used here is at a similar level to that used by Ohanessian et al. for their study of biomimetic Zn complexes.[16] In this study, it was demonstrated that reliable results in terms of geometry and chemical accuracy could be reached by simply performing a HF geometry optimization, followed by a B3LYP energy calculation with a larger basis set. In this work, we used the M06-2X functional (instead of the popular B3LYP) as it also includes dispersion correction (see the Supporting Information for details). Changing the configuration of the hydrate and reversing the propeller twist around the tertiary amine in the complex revealed a structure that was essentially the same in energy as the initial conformation (within 1 kcal mol^−1^). By introducing methyl 4-nitrophenyl phosphate to the Zn ion in place of the water molecule, we find similar low-energy conformations where the uncoordinated oxygen atom is within 4.1 Å of the phosphate, and close to in-line with the leaving group (164°) as shown in Figure 2. Thus, the participation of the noncoordinated oxygen atom as a nucleophile is geometrically feasible. Performing a transition-state optimization for the nucleophile attack reaction of the noncoordinated oxygen atom revealed a transition state which was characterized by frequency calculations, and the minimum-energy path connecting reactants to products through this transition state was evaluated by calculating the intrinsic reaction coordinate to confirm this is a viable pathway for the phosphoryl transfer reaction (see the Supporting Information for details).

A direct test of this proposal is not practical in aqueous solution, as the two hydroxy groups cannot be distinguished. If the reaction is carried out in dry methanol, the two sites are differentiated as the noncoordinated position is methylated (as illustrated by the crystal structure of **4′** in Figure 2). Thus, we compared the reactivity in methanol solution (which is known to provide a large rate acceleration for many zinc complexes acting on phosphate esters).[17] Similarly to the aqueous reactions, we observe a bell-shaped dependence on the p*K*_a_ value, thus showing that the monodeprotonated species is the dominant active form for **2**, **3**, and **4′** (Figure 3). Similar to the reports of Brown and co-workers, we observe that in methanol, the rate of reaction of BNPP in the presence of **2** and **3** is much greater, with increases in the limiting second-order rate constants of about 1000-fold.[17] However, the reactivity of **4′** is barely modified compared to that of **4** (ca. threefold increase), and so in methanol the maximal rate in the presence of **4′** is 300-fold slower than in the presence of **3**. We interpret this observation as confirming the analysis of Mancin and co-workers, and that the activity of the coordinated oxyanion is severely reduced because of the inductive effect of the adjacent methoxy group.

**Figure 3:**
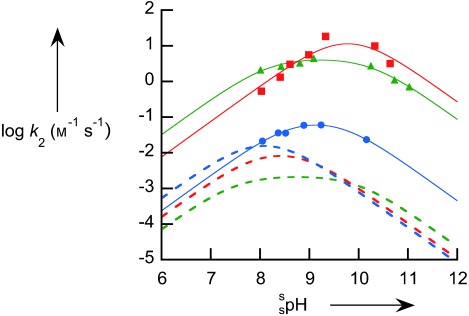
pH rate profile for the cleavage of BNPP catalyzed by 2 (green triangles), by 3 (red squares), and by 4′ (blue circles) at 25 °C in anhydrous methanol, [buffer]=0.05 m). Solid lines are from fitting Equation (1) to the data,[13] and the dashed lines illustrate the corresponding reactivity in water.

Overall, the incorporation of an aldehyde functionality has allowed conversion of a stoichiometric reagent into a catalytic complex. The effect of methylating selected positions provides approximately a tenfold enhancement in the activity of the parent complex. Unexpectedly, the use of an aldehyde hydrate as a nucleophile is not accompanied by a decrease in reactivity relative to that of an alcohol, thus leading us to propose that this system reacts through a nucleophile which is not coordinated to the metal ion. This proposal contrasts with the general strategy of designing this type of complex, and suggests that incorporating non metal-ion bound nucleophiles into a ligand may be a productive route to creating more effective complexes by avoiding nucleophile deactivation through metal ion coordination, thus enhancing the Lewis acidity of the metal ion in the active tautomeric form, and allowing more favorable geometries for the delivery of the nucleophile to the coordinated substrate. We note that the mechanisms assumed for RNA coordinated to metal ion complexes follow this mechanistic course (a noncoordinated alkoxy nucleophile with a high p*K*_a_ value), so substituting the role of the 2′OH with a carbonyl hydrate site on the ligand provides a strategy for designing complexes effective for DNA hydrolysis as well. Finally, in considering the active sites of sulfatases and phosphonohydrolases, which use formyl glycine as a nucleophile, a metal ion is present and coordinated to the hydrated aldehyde. Our data suggest that the most catalytically active tautomer will be the one in which the noncoordinated hydroxy is ionized, thus furnishing a more effective Lewis acid and more reactive nucleophile.

## Experimental Section

Kinetic experiments were carried out at 25 °C, either in water with 50 mm buffer at 0.1 m ionic strength (NaNO_3_) or in anhydrous methanol with 50 mm buffer and monitored using UV/Vis spectroscopy to measure the change in absorbance at 400 nm (water) or 320 nm (methanol). In water, a typical experiment was initiated by the addition of 0.5 mL of 4 mm BNPP (in 50 mm buffer at 0.1 m ionic strength and 25 °C) to a 1 mL cuvette containing 0.5 mL of a solution of Zn complex (in 50 mm buffer at 0.1 m ionic strength) which had also been equilibrated at 25 °C. In anhydrous methanol, 50 μL of 1 mm BNPP (in anhydrous methanol) was added to a 1 mL cuvette containing 0.95 mL of a solution of Zn complex (in 50 mm buffer) and equilibrated at 25 °C. See the Supporting Information for details of synthesis and characterization of ligands, kinetic and potentiometric data, product analyses, computational methods, and the CIF for **4′**.
