# Supplementary material for: Catalytic Zinc Complexes for Phosphate Diester Hydrolysis
Source: Angew Chem Int Ed Engl. 2014 Jun 11;53(31):8246–50. doi: 10.1002/anie.201400335 (PMC4140542; doi:10.1002/anie.201400335)
Supplement: Supplementary file 1 [file anie0053-8246-sd1.pdf]

Angewandte Chemie
Eine Zeitschrift der Gesellschaft Deutscher Chemiker

Supporting Information

© Wiley-VCH 2014

69451 Weinheim, Germany

Catalytic Zinc Complexes for Phosphate Diester Hydrolysis**

*Emmanuel Y. Tirel, Zoë Bellamy, Harry Adams, Vincent Lebrun, Fernanda Duarte, and Nicholas H. Williams**

anie_201400335_sm_miscellaneous_information.pdf

Supporting information

Table of content

Synthesis 2

3-(bis ((6-methylpyridin-2-yl) methyl) amino) propan-1-ol (L2)[2] 2

3-(bis ((6-methylpyridin-2-yl)methyl)amino)-2,2-dimethylpropan-1-ol (L3) 2

3-(bis ((6-methylpyridin-2-yl) methyl) amino)-2, 2-dimethylpropan-1-al (L4) 3

3-(bis((6-methylpyridin-2-yl)methyl)amino)-2,2-dimethylpropane (L5) 3

Zn complex of L4 [4'.$(NO_3)_2$] 3

Crystallographic details for Zn complex of L4 [4'$(NO_3)_2$] 4

Kinetic data for Zn complex 2. 5

Titration data and speciation curves of L2 and Zn complex 2. 5

Kinetic data for Zn complex 3. 6

Titration data and speciation curves of L3 and Zn complex 3. 8

Kinetic data for Zn complex 4. 9

Titration data and speciation curves of L4 and Zn complex 4. 12

Product analysis of the cleavage of BNPP by Zn complexes 3, 4 and 5. 13

Cleavage of BNPP in methanol by Zn complexes 2, 3 and 4'. 14

Computational Methods. 17

Computational Results. 18

References 38

Synthesis

6-Methyl-2-pyridinemethanol was purchased from Maybridge and used without further purification. It was converted to 6-methyl-2-(bromomethyl)-pyridine according to the method of Lippard *et al.*[1]

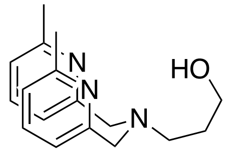

L2

3-(bis ((6-methylpyridin-2-yl) methyl) amino) propan-1-ol (L2)[2]

To a solution of 6-methyl-2-(bromomethyl)-pyridine[1] (535 mg, 2.89 mmol) in dry DMF (30 mL) were added 3-amino-1-propanol (100 µL, 1.32 mmol) and then *N*,*N*-Diisopropylethylamine (690 µL, 3.96 mmol). The reaction was stirred for 16 h at 70 °C. The solution was poured into 40 mL H_2O and the organic component was extracted with CH_2Cl_2 (3 × 30 mL) and dried over $MgSO_4$. The solvent was removed under reduce pressure and the product was purified using silica gel chromatography (EtOAc : MeOH - 9:1) to yield 350 mg of **L2**[2] as a yellow oil (92%).

δ_H (400 MHz, $CDCl_3$): 7.53 (2 H, t, 7.5, Py-H), 7.23 (2 H, d, 7.5, Py-H), 7.00 (2 H, d, 7.4, Py-H), 3.75 (4 H, s, Py-CH_2), 2.73 (2 H, t, 7.1, CH_2-O), 2.56 (2 H, s, CH_2-N), 2.55 (6 H, s, CH_3), 1.70 (2 H, qn, 7.6, C-CH_2-C); δ_C (101 MHz, $CDCl_3$): 157.8 (Py-N), 136.9 (Py), 121.7 (Py), 120.0 (Py), 62.0 (C-O), 59.8 (C-N), 52.7 (C-N), 28.7 (CH_2), 24.2 (CH_3); HRMS (TOF mode) calculated for $C_{17}H_{24}N_3O$ (MH^+), 286.1919, found 286.1915

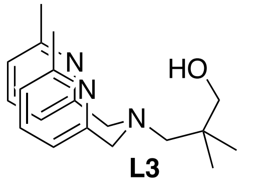

L3

3-(bis ((6-methylpyridin-2-yl)methyl)amino)-2,2-dimethylpropan-1-ol (L3)

To a solution of 3-amino-2,2-dimethyl-1-propanol (0.350 g, 3.39 mmol) in dry DMF (50 mL) were added 6-methyl-2-(bromomethyl)-pyridine[1] (1.4 g, 7.57 mmol) and then *N*,*N*-Diisopropylethylamine (1.80 mL, 10.2 mmol). The reaction was stirred for 16 h at 70 °C. The solution was poured into 60 mL of H_2O and the organic component was extracted with CH_2Cl_2 (3 × 40 mL) and dried over $MgSO_4$. The solvent was removed under reduce pressure and the product was purified using silica gel chromatography (hexane: EtOAc - 1:9) to yield 0.73 g of **L3** as a pale yellow oil (68%).

δ_H (400 MHz, $CDCl_3$): 7.52 (2 H, t, 7.5, Py-H), 7.20 (2 H, d, 7.5, Py-H), 7.01 (2 H, d, 7.4, Py-H), 3.85 (4 H, s, Py-CH_2), 3.35 (2 H, s, CH_2-O), 2.60 (s, 2H, CH_2-N), 2.59 (s, 6 H, CH_3), 0.91 (s, 6H, CH_3); δ_C (101 MHz, $CDCl_3$): 158.4 (Py-N), 157.7 (Py-N), 136.7 (Py), 121.6 (Py), 120.2 (Py), 71.3 (C-O), 64.3 (C-N), 62.7 (C-N), 36.8 (C-CH_3), 24.8 (CH_3), 24.3 (CH_3); HRMS (TOF mode) calculated for $C_{19}H_{28}N_3O$ (MH^+), 314.2232, found 314.2242

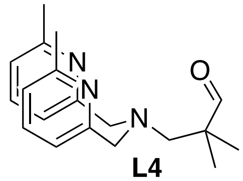

L4

3-(bis ((6-methylpyridin-2-yl) methyl) amino)-2, 2-dimethylpropan-1-al (L4)
A solution of Dess Martin periodinane (0.83 g, 1.97 mmol) in 20 mL dry CH_2Cl_2 was added to a solution of 3-(bis((6-methylpyridin-2-yl)methyl)amino)-2,2-dimethylpropan-1-ol (**L3**) (0.56 g, 1.79 mmol) in 20 mL of dry CH_2Cl_2 at 0 °C under a nitrogen atmosphere. After 2 h at room temperature, 40 mL of H_2O was added to the reaction mixture and the organic component was extracted with CH_2Cl_2 (3 × 20 mL). The extract was dried over $MgSO_4$ and the solvent removed under reduced pressure. The product was purified using silica gel chromatography (EtOAc) to yield 0.36 g of **L4** as a pale yellow oil (64%).

δ_H (400 MHz, $CDCl_3$): 9.26 (1 H, s, O=C-H), 7.56 (2 H, t, 7.5, Py-H), 7.29 (2 H, d, 7.5, Py-H), 7.05 (2 H, d, 7.4, Py-H), 3.78 (4 H, s, Py-CH_2), 2.91 (2 H, s, CH_2-N), 2.55 (6 H, s, CH_3), 1.01 (6 H, s, CH_3); δ_C (101 MHz, $CDCl_3$): 206.2 (C=O), 158.8 (Py-N), 157.5 (Py-N), 136.7 (Py), 121.6 (Py), 120.0 (Py), 62.1 (C-N), 61.5 (C-N), 41.1 (C-C), 24.3 (CH_3), 20.4 (CH_3); HRMS (TOF mode) calculated for $C_{19}H_{26}N_3O$ (MH^+), 312.2076, found 312.2068

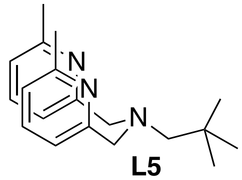

L5

3-(bis((6-methylpyridin-2-yl)methyl)amino)-2,2-dimethylpropane (L5)
To a solution of 2,2-dimethylpropan-1-amine (0.60 mL, 0.44 g, 4.9 mmol) in dry DMF (50 mL) was added 6-methyl-2-(bromomethyl)-pyridine[1] (2.00 g, 10.8 mmol) and K_2CO_3 (1.49 g, 10.8 mmol). The solution was stirred at 40 °C for 16 h. The organic component was extracted into CH_2Cl_2 (3 × 40 mL), the extract dried over $MgSO_4$, filtered and the solvent was removed under reduced pressure. The product was purified using silica gel chromatography (Hexane : EtOAc - 10:1) to yield 1.28 g of **L5** as a pale yellow solid (88%).

δ_H (400 MHz, $CDCl_3$): 7.57 (2 H, t, 7.5, Py-H), 7.41 (2 H, d, 7.5, Py-H), 7.00 (2 H, d, 7.4, Py-H), 3.85 (4 H, s, Py-CH_2), 2.55 (6 H, s, CH_3), 2.54 (2 H, s, CH_2-N), 0.80 (9 H, s, CH_3); δ_C (101 MHz, $CDCl_3$): 159.9 (Py-N), 157.4 (Py-N), 136.4 (Py), 121.2 (Py), 119.9 (Py), 66.6 (C-N), 63.1 (C-N), 33.1 (q C-C), 28.2 (CH_3), 24.5 (CH_3); HRMS (TOF mode) calculated for $C_{19}H_{28}N_3$ (MH^+), 298.2283, found 298.2278

Zn complex of L4 [4'.$(NO_3)_2$]
A solution of $Zn(NO_3)_2(H_2O)_6$ (24 mg, 0.081 mmol in 2 mL MeOH) was added to a solution of **L4** (23 mg, 0.074 mmol in 2 mL MeOH). After 10 min stirring, the solution was filtered and the volume reduced to 1 mL. Et_2O was introduced by slow diffusion and colourless crystals of X-ray quality were collected after two days (27 mg, 70% yield).

Crystallographic details for Zn complex of L4 [4'$(NO_3)_2$]

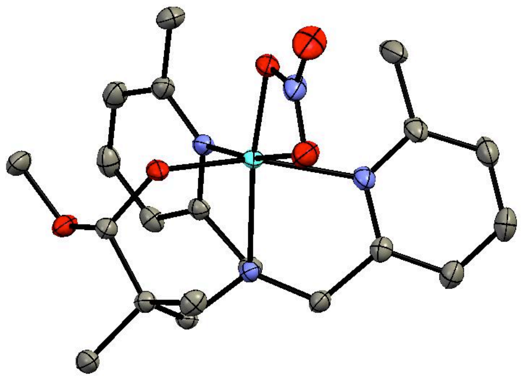

Figure S1. Representation of the X-ray crystal structure of 4'-$(NO_3)_2$ (50% probability ellipsoids; hydrogen atoms and non-coordinated nitrate omitted for clarity). Selected bond distances: Zn(1)-N(1) = 2.131Å; Zn(1)-N(2) = 2.149 Å; Zn(1)-N(3) = 2.062 Å; Zn(1)-O(1) = 2.534 Å; Zn(1)-O(2) = 2.045 Å; Zn(1)-O(3) = 2. 076 Å

Empirical formula	$C_{20} H_{29} N_5 O_8 Zn$	
Formula weight	532.85	
Temperature	100(2) K	
Wavelength	0.71073 Å	
Crystal system	Orthorhombic	
Space group	Pbca	
Unit cell dimensions	a = 14.9370(4) Å	α = 90°.
	b = 16.1280(4) Å	β = 90°.
	c = 19.7011(5) Å	γ = 90°.
Volume	4746.1(2) $Å^3$	
Z	8	
Density (calculated)	1.491 Mg/m^3	
Absorption coefficient	1.090 mm^{-1}	
F(000)	2224	
Crystal size	0.32 x 0.21 x 0.18 mm^3	
Theta range for data collection	2.07 to 27.87°.	
Index ranges	-19<=h<=19, -21<=k<=21, -25<=l<=25	
Reflections collected	101826	
Independent reflections	5588 [R(int) = 0.0495]	
Completeness to theta = 27.87°	98.7 %	
Absorption correction	Semi-empirical from equivalents	
Max. and min. transmission	0.8280 and 0.7218	
Refinement method	Full-matrix least-squares on F^2	
Data / restraints / parameters	5588 / 0 / 315	
Goodness-of-fit on F^2	0.887	
Final R indices [I>2sigma(I)]	R1 = 0.0310, wR2 = 0.0910	
R indices (all data)	R1 = 0.0362, wR2 = 0.0963	
Largest diff. peak and hole	0.520 and -0.412 e.$Å^{-3}$	

Kinetic data for Zn complex 2.

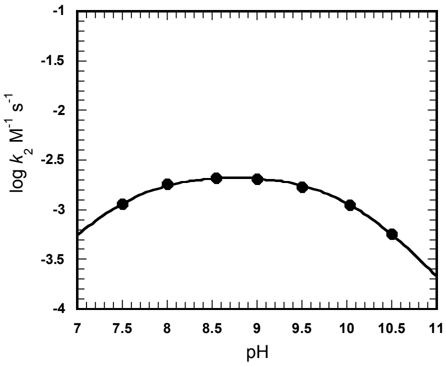

pH	k_2 M^{-1} s^{-1}	log(k_2) M^{-1} s^{-1}
7.50	1.13x10^{-3}	-2.95
8.00	1.81x10^{-3}	-2.74
8.55	2.07x10^{-3}	-2.68
9.00	2.04x10^{-3}	-2.69
9.50	1.71x10^{-3}	-2.77
10.03	1.12x10^{-3}	-2.95
10.50	5.68x10^{-4}	-3.25

Figure S2. pH rate profile for the transesterification of BNPP catalysed by Zn complex 2 (50 mM buffer; 0.1 M ionic strength ($NaNO_3$); 25 °C). The solid line is the least squares fit of equation 1 (main text) to the data.

Titration data and speciation curves of L2 and Zn complex 2.

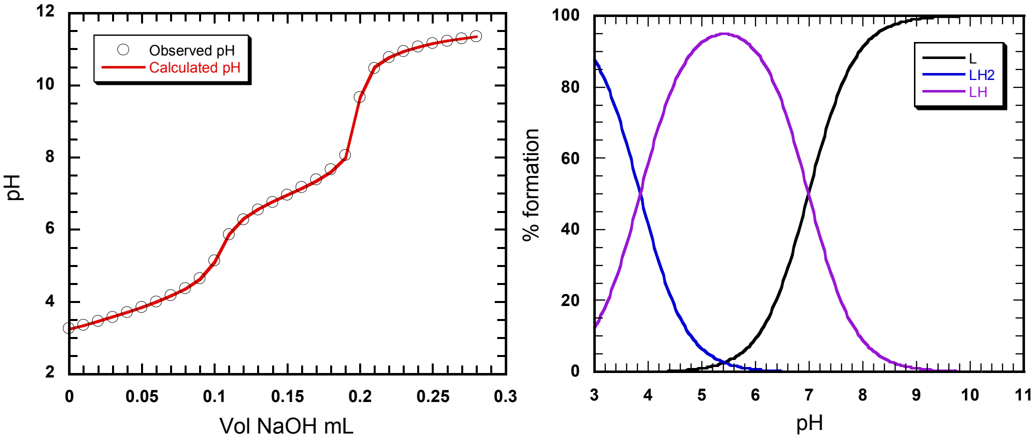

Figure S3. left: titration of 10 mL of 2 mM L2 and 4 mM HCl with 10 µL aliquots of 0.2 mM NaOH; right: speciation diagram of 1mM L2. The theoretical fit and speciation were carried out with Hyperquad and HySS software[3] and gave pK_as of 3.85 and 6.99.

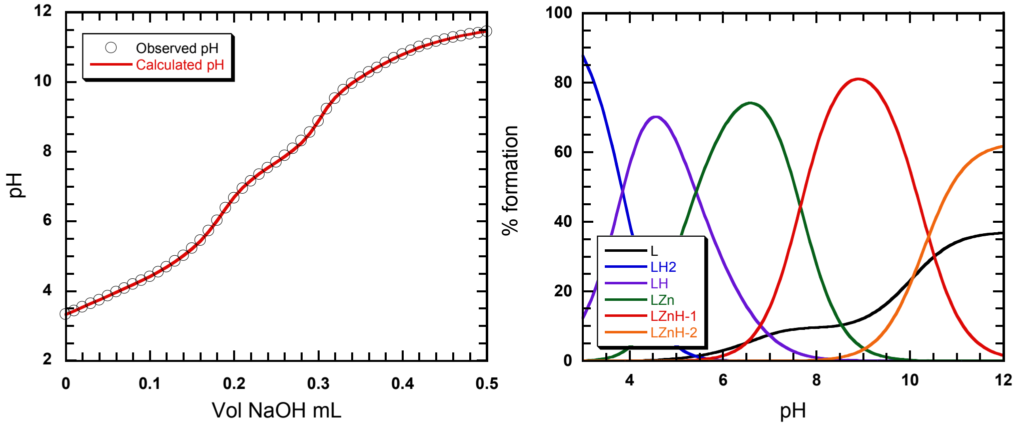

Figure S4. left: titration of 10 mL of 2 mM Zn complex 2 and 4 mM HCl with 10 µL aliquots of 0.2 mM NaOH; right: speciation diagram of 1 mM 2. The theoretical fit and speciation were carried out with Hyperquad and HySS software[3] using formation constants for Zn(OH) ($10^{-7.84}$) and $Zn(OH)_2$ ($10^{-16.86}$) taken from literature.[4] The fit gives pK_as for the Zn complex 2 of 7.84 and 9.02, and log K_d as 4.85.

Kinetic data for Zn complex 3.

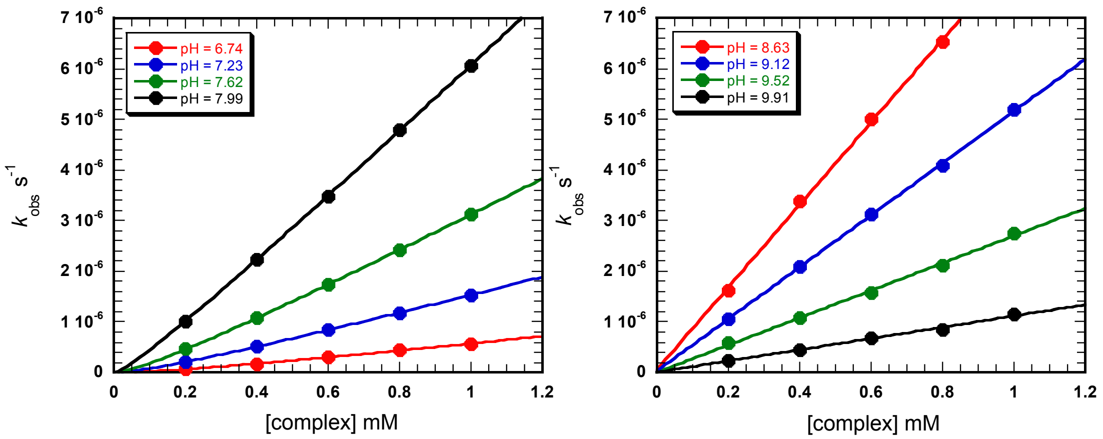

Figure S5. Plots of observed rate constants for the transesterification of BNPP (2 mM) against concentration of Zn complex 3. Left: pH from 6.74 to 7.99; right: pH from 8.63 to 9.91. The solid lines are the least squares fit of equation S1 to the data.

Observed rate constants (k_{obs}) were obtained by the initial rate method over the first 30 minutes of the reaction. Equation S1 is derived from Scheme S1, and fitted to the data in Tables S1 to S8 to give the values of k_2 used in Figure S5.

$$k_{obs} = k_2 \times \frac{1}{2}\{(K_d + [Zn]_T + [L]_T) - \sqrt{(K_d + [Zn]_T + [L]_T)^2 - 4[Zn]_T[L]_T}\} \qquad \text{Equation S1}$$

$$Zn + L \underset{K_d}{\rightleftharpoons} ZnL \xrightarrow[k_2]{BNPP} products$$

Scheme S1

[**3**] mM	k_{obs} s^{-1} at pH 6.74	k_{obs} s^{-1} at pH 7.23	k_{obs} s^{-1} at pH 7.62	k_{obs} s^{-1} at pH 7.99
0.2	6.6±0.4×10^{-8}	2.04±0.01×10^{-7}	4.59±0.02×10^{-7}	9.9±0.2×10^{-7}
0.4	1.83±0.08×10^{-7}	5.11±0.02×10^{-7}	1.074±0.002×10^{-6}	2.233±0.007×10^{-6}
0.6	3.03±0.03×10^{-7}	8.42±0.02×10^{-7}	1.722±0.009×10^{-6}	3.479±0.005×10^{-6}
0.8	4.50±0.01×10^{-7}	1.183±0.002×10^{-6}	2.42±0.01×10^{-6}	4.780±0.004×10^{-6}
1.0	5.72±0.06×10^{-7}	1.530±0.001×10^{-6}	3.10±0.02×10^{-6}	6.072±0.005×10^{-6}
[**3**] mM	k_{obs} s^{-1} at pH 8.63	k_{obs} s^{-1} at pH 9.12	k_{obs} s^{-1} at pH 9.52	k_{obs} s^{-1} at pH 9.91
0.2	1.63±0.01×10^{-6}	1.070±0.007×10^{-6}	5.7±0.1×10^{-7}	2.34±0.05×10^{-7}
0.4	3.35±0.02×10^{-6}	2.084±0.002×10^{-6}	1.07±0.01×10^{-6}	4.50±0.07×10^{-7}
0.6	5.023±0.02×10^{-6}	3.105±0.009×10^{-6}	1.60±0.03×10^{-6}	6.5±0.4×10^{-7}
0.8	6.61±0.08×10^{-6}	4.12±0.02×10^{-6}	2.13±0.01×10^{-6}	8.1±0.3×10^{-7}
1.0	8.28±0.08×10^{-6}	5.22±0.04×10^{-6}	2.78±0.02×10^{-6}	1.11±0.04×10^{-6}

Table S1. Observed rate constants for the cleavage of BNPP in the presence of Zn complex 3 (50 mM buffer; 0.1 M ionic strength ($NaNO_3$); 25 °C)

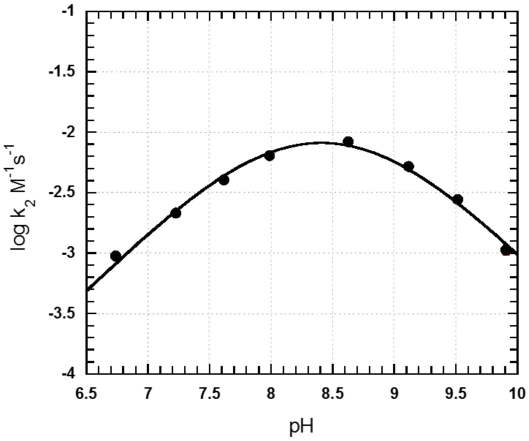

pH	k_2 M^{-1} s^{-1}	Standard deviation	$\log(k_2)$ M^{-1} s^{-1}
6.74	$8.83x10^{-4}$	$0.05x10^{-3}$	-3.05
7.23	$2.12x10^{-3}$	$0.03x10^{-3}$	-2.67
7.62	$4.00x10^{-3}$	$0.03x10^{-3}$	-2.40
7.99	$7.11x10^{-3}$	$0.05x10^{-3}$	-2.15
8.63	$8.28x10^{-3}$	$0.07x10^{-3}$	-2.08
9.12	$5.17x10^{-3}$	$0.05x10^{-3}$	-2.29
9.52	$2.74x10^{-3}$	$0.08x10^{-3}$	-2.56
9.91	$1.05x10^{-3}$	$0.06x10^{-3}$	-2.98

Figure S6. pH rate profile for the transesterification of BNPP catalysed by Zn complex 3 (50 mM buffer; 0.1 M ionic strength ($NaNO_3$); 25 °C). The solid line is the least squares fit of equation 1 (main text) to the data.

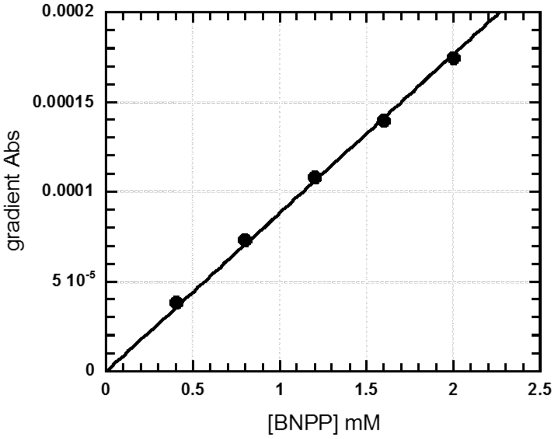

[BNPP] mM	gradient Abs
0.4	3.84 e-5
0.8	7.33e-5
1.2	1.08e-4
1.6	1.40e-4
2	1.74e-4

Figure S7. Plots of the dependence of the change in absorbance at 400 nm on BNPP concentration in the presence of 1 mM Zn complex 3 (25 °C, 50 mM buffer, 0.1 M ionic strength ($NaNO_3$), pH = 8.1), demonstrating first order dependence of rate on [BNPP]. The solid line is the linear least squares fit to the data.

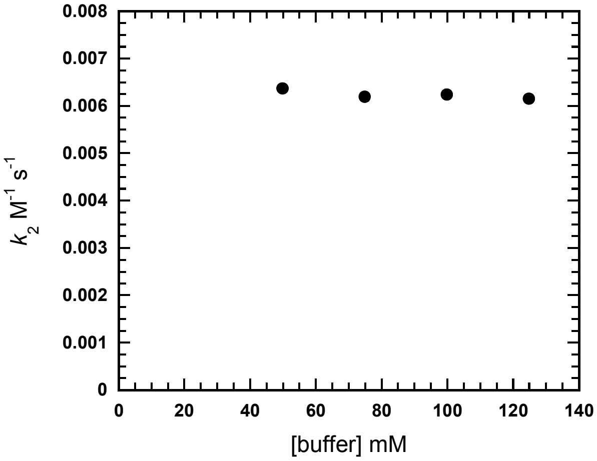

[buffer] mM	k_2 M^{-1} s^{-1}
50	$6.35±0.02 x 10^{-3}$
75	$6.18±0.06 x 10^{-3}$
100	$6.22±0.01 x 10^{-3}$
125	$6.14±0.03 x 10^{-3}$

Figure S8. Plots of the dependence of the limiting second order rate constant k_2 for the transesterification of BNPP (2 mM) on buffer concentration in the presence of Zn complex 3 (25 °C, 0.1 M ionic strength ($NaNO_3$), pH = 8.1), demonstrating the absence of buffer catalysis.

Titration data and speciation curves of L3 and Zn complex 3.

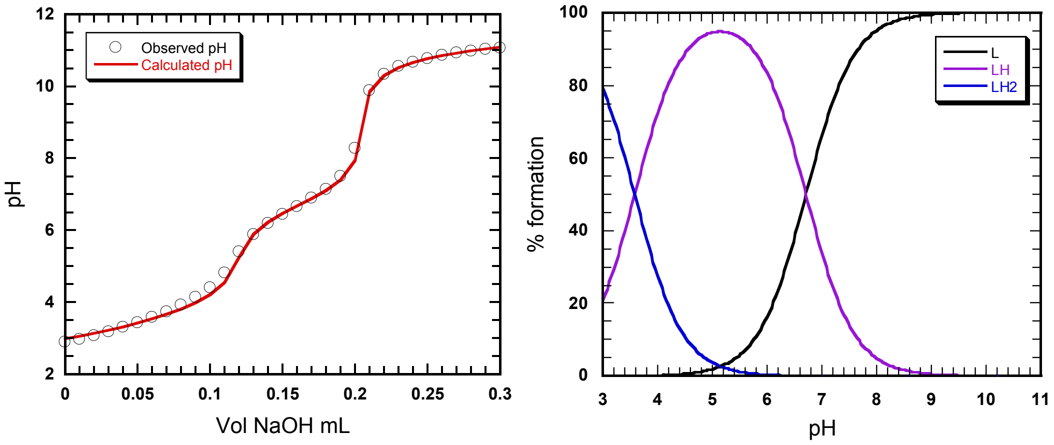

Figure S9. left: titration of 10 mL of 2 mM L3 and 4 mM HCl with 10 µL aliquots of 0.2 mM NaOH; right: speciation diagram of 1mM L3. The theoretical fit and speciation were carried out with Hyperquad and HySS software[3] and gave pK_as of 3.58 and 6.71.

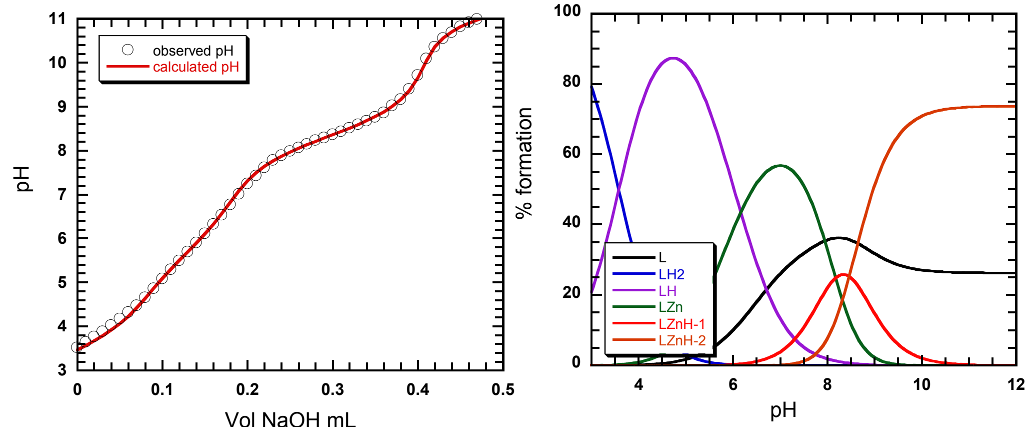

Figure S10. left: titration of 10 mL of 2 mM Zn complex 3 and 4 mM HCl with 10 µL aliquots of 0.2 mM NaOH; right: speciation diagram of 1 mM 3. The theoretical fit and speciation were carried out with Hyperquad and HySS software[3] using formation constants for Zn(OH) ($10^{-7.84}$) and $Zn(OH)_2$ ($10^{-16.86}$) taken from literature.[4] The fit gives pK_as for the Zn complex 2 of 8.17 and 8.46, and log K_d as 3.80.

Kinetic data for Zn complex 4.

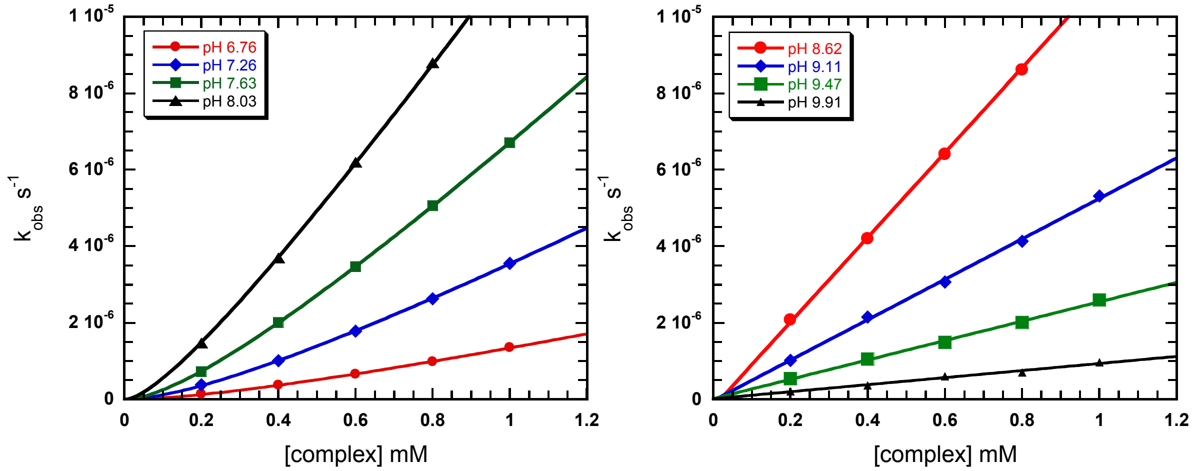

Figure S11. Plots of observed rate constants for the transesterification of BNPP (2 mM) against concentration of Zn complex 4. Left: pH from 6.76 to 8.03; right: pH from 8.62 to 9.91. The solid lines are the least squares fit of equation S1 to the data.

Observed rate constants (k_{obs}) were obtained by the initial rate method over the first 30 minutes of the reaction. Equation S1 derived from Scheme S1 was fitted to the data in Tables S10 to S17 to give the values of k_2 used in Figure S12.

[**4**] mM	k_{obs} s^{-1} at pH 6.76	k_{obs} s^{-1} at pH 7.27	k_{obs} s^{-1} at pH 7.63	k_{obs} s^{-1} at pH 8.03
0.2	$1.40{\pm}0.02{\times}10^{-7}$	$3.76{\pm}0.07{\times}10^{-7}$	$7.33{\pm}0.09{\times}10^{-7}$	$1.46{\pm}0.08{\times}10^{-7}$
0.4	$3.33{\pm}0.3{\times}10^{-7}$	$1.001{\pm}0.001{\times}10^{-6}$	$2.004{\pm}0.001{\times}10^{-6}$	$3.71{\pm}0.01{\times}10^{-6}$
0.6	$6.55{\pm}0.01{\times}10^{-7}$	$1.778{\pm}0.007{\times}10^{-6}$	$3.49{\pm}0.01{\times}10^{-6}$	$6.21{\pm}0.01{\times}10^{-6}$
0.8	$9.77{\pm}0.04{\times}10^{-7}$	$2.630{\pm}0.004{\times}10^{-6}$	$5.08{\pm}0.03{\times}10^{-6}$	$8.79{\pm}0.01{\times}10^{-6}$
1.0	$1.35{\pm}0.002{\times}10^{-6}$	$3.546{\pm}0.007{\times}10^{-6}$	$6.74{\pm}0.03{\times}10^{-6}$	$1.139{\pm}0.002{\times}10^{-5}$
[**4**] mM	k_{obs} s^{-1} at pH 8.62	k_{obs} s^{-1} at pH 9.11	k_{obs} s^{-1} at pH 9.47	k_{obs} s^{-1} at pH 9.91
0.2	$1.98{\pm}0.09{\times}10^{-6}$	$1.01{\pm}0.01{\times}10^{-6}$	$5.30{\pm}0.06{\times}10^{-7}$	$2.00{\pm}0.07{\times}10^{-7}$
0.4	$4.14{\pm}0.05{\times}10^{-6}$	$2.12{\pm}0.04{\times}10^{-6}$	$1.055{\pm}0.004{\times}10^{-6}$	$3.52{\pm}0.1{\times}10^{-7}$
0.6	$6.27{\pm}0.1{\times}10^{-6}$	$3.09{\pm}0.03{\times}10^{-6}$	$1.53{\pm}0.04{\times}10^{-6}$	$5.4{\pm}0.5{\times}10^{-7}$
0.8	$8.36{\pm}0.1{\times}10^{-6}$	$4.13{\pm}0.01{\times}10^{-6}$	$1.99{\pm}0.01{\times}10^{-6}$	$6.80{\pm}0.1{\times}10^{-7}$
1.0	$1.08{\pm}0.01{\times}10^{-5}$	$5.28{\pm}0.03{\times}10^{-6}$	$2.60{\pm}0.01{\times}10^{-6}$	$9.56{\pm}0.08{\times}10^{-7}$

Table S2. Observed rate constants for the cleavage of BNPP in the presence of Zn complex 4 (50 mM buffer; 0.1 M ionic strength ($NaNO_3$); 25 °C)

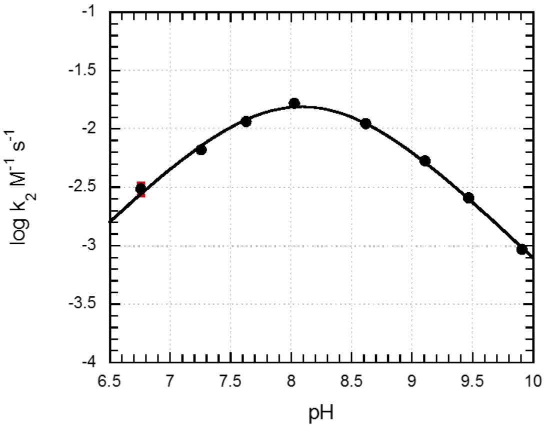

pH	k_2 M^{-1} s^{-1}	Standard deviation	log(k_2) M^{-1} s^{-1}
6.76	3.01×10^{-3}	0.3×10^{-3}	-2.52
7.26	6.57×10^{-3}	0.2×10^{-3}	-2.18
7.63	1.15×10^{-2}	0.005×10^{-2}	-1.94
8.03	1.64×10^{-2}	0.02×10^{-2}	-1.79
8.62	1.10×10^{-2}	0.02×10^{-2}	-1.96
9.11	5.27×10^{-3}	0.08×10^{-3}	-2.28
9.47	2.54×10^{-3}	0.07×10^{-3}	-2.60
9.91	9.20×10^{-4}	0.6×10^{-4}	-3.04

Figure S12. pH rate profile for the transesterification of BNPP catalysed by Zn complex 4 (50 mM buffer; 0.1 M ionic strength ($NaNO_3$); 25 °C). The solid line is the least squares fit of equation 1 (main text) to the data.

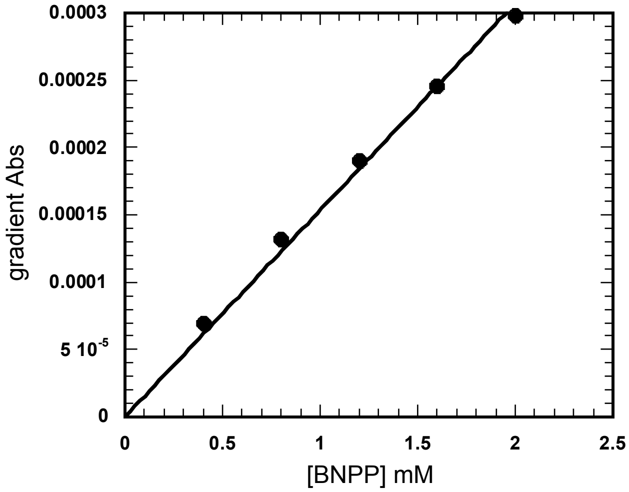

[BNPP] mM	gradient Abs
0.4	6.90×10^{-5}
0.8	1.32×10^{-4}
1.2	1.90×10^{-4}
1.6	2.46×10^{-4}
2	2.98×10^{-4}

Figure S13. Plot of the dependence of the change in absorbance at 400 nm on BNPP concentration in the presence of 1 mM Zn complex 4 (25 °C, 50 mM buffer, 0.1 M ionic strength ($NaNO_3$), pH = 8.0), demonstrating first order dependence of rate on [BNPP].

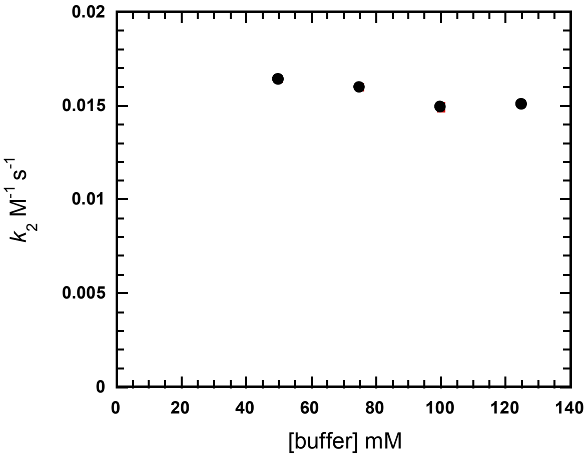

[buffer] mM	k_2 M^{-1} s^{-1}
50	$1.64 \pm 0.01 \times 10^{-2}$
75	$1.60 \pm 0.01 \times 10^{-2}$
100	$1.49 \pm 0.02 \times 10^{-2}$
125	$1.51 \pm 0.01 \times 10^{-2}$

Figure S14. Plots of the dependence of the limiting second order rate constant k_2 for the transesterification of BNPP (2 mM) on buffer concentration in the presence of Zn complex 4 (25 °C, 0.1 M ionic strength ($NaNO_3$), pH = 8.1), demonstrating the absence of buffer catalysis.

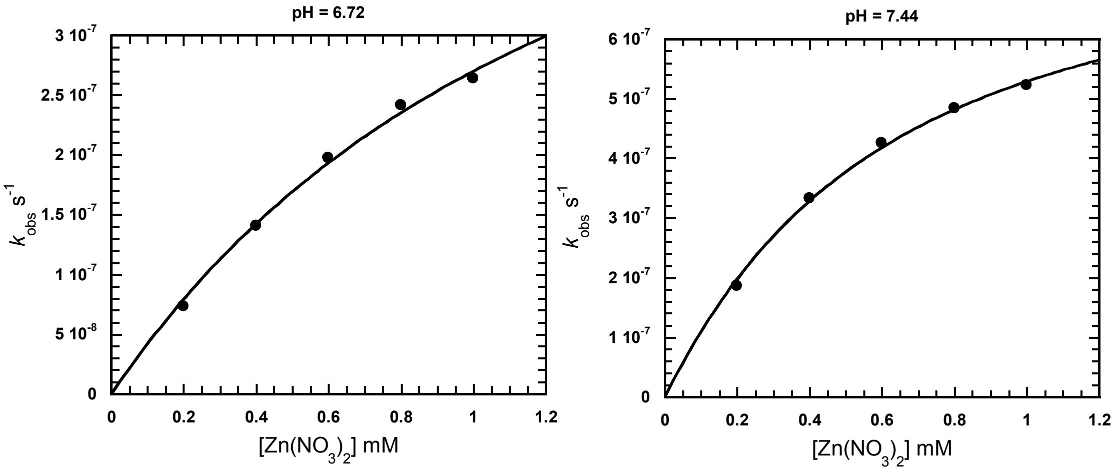

Figure S15. Dependence of k_{obs} on the Zn:ligand ratio; left: pH 6.72; right: pH 7.44. [L4] = 0.2 mM; 0.1 M ionic strength ($NaNO_3$); 25 °C. Solid lines are from linear least squares fitting equation S2 (derived from scheme S1) to the data and yield to K_d^{app} = 1.2±0.3 mM and k_2 = 3.1±0.1 × 10^{-3} M^{-1} s^{-1} at pH = 6.72 and K_d^{app}= 0.48±0.06 mM and k_2 = 4.1±0.1 × 10^{-3} M^{-1} s^{-1} at pH = 7.44.

$$k_{obs} = k_2 \times \frac{1}{2}\{(K_d^{app} + [\mathrm{Zn}]_\mathrm{T} + [\mathrm{L4}]_\mathrm{T}) - \sqrt{(K_d^{app} + [\mathrm{Zn}]_\mathrm{T} + [\mathrm{L4}]_\mathrm{T})^2 - 4[\mathrm{Zn}]_\mathrm{T}[\mathrm{L4}]_\mathrm{T}}\,\} \qquad \text{Equation S2}$$

The values of the conditional dissociation constant K_d^{app} differ by a factor of ~2.6 fold, which is in good agreement with variation expected at these pHs based on the pK_a of **L4** (6.91) and the first pK_a of the Zn complex of **4** (8.09) established by potentiometric titration. The limiting second rate constants agree well with the data obtained at constant Zn:ligand ratio; the K_d values are about 4 fold greater than obtained from potentiometric titration (0.12 mM).

Titration data and speciation curves of L4 and Zn complex 4.

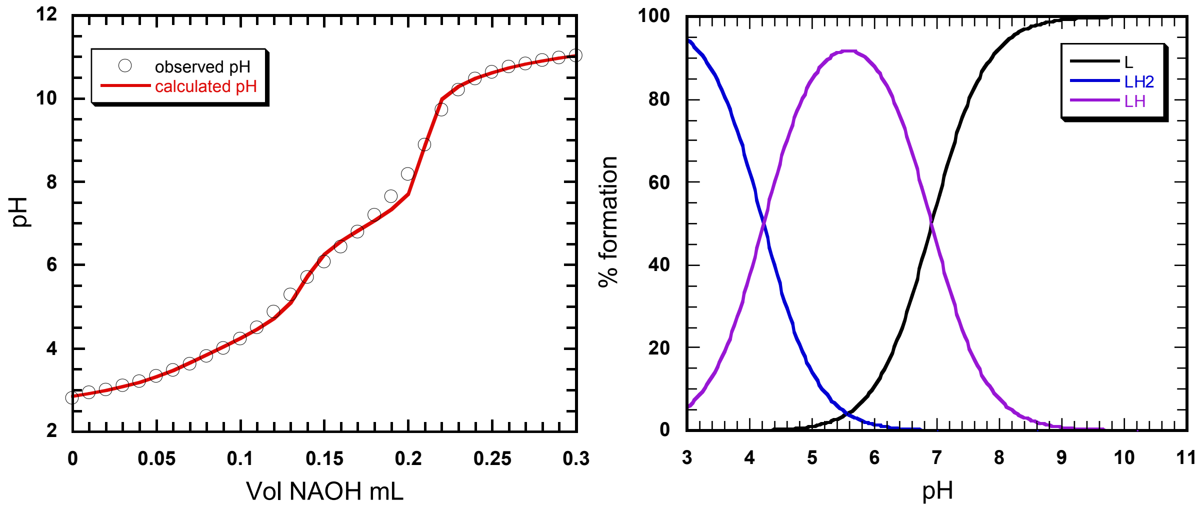

Figure S16. left: titration of 10 mL of 2 mM L4 and 4 mM HCl with 10 µL aliquots of 0.2 mM NaOH; right: speciation diagram of 1mM L4. The theoretical fit and speciation were carried out with Hyperquad and HySS software[3] and gave pK_as of 4.23 and 6.91.

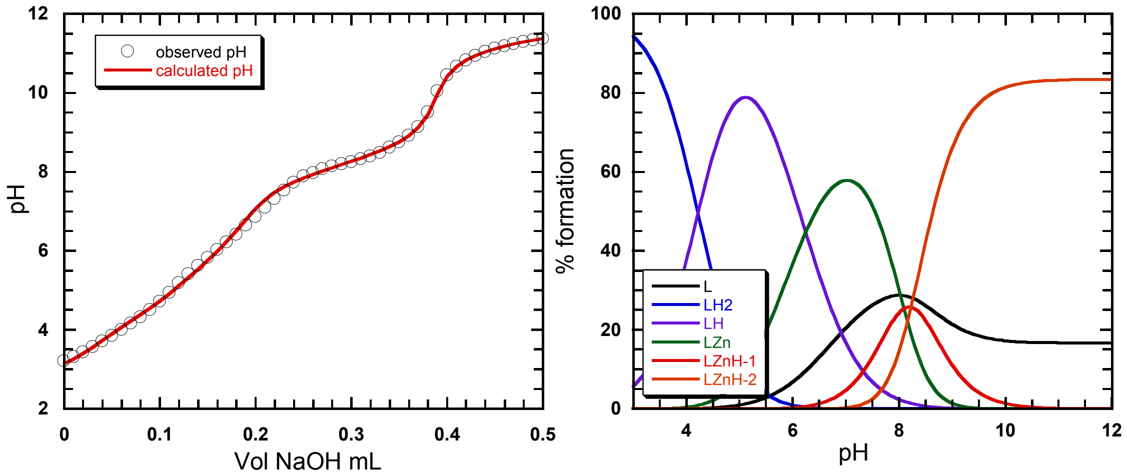

Figure S17. left: titration of 10 mL of 2 mM Zn complex 4 and 4 mM HCl with 10 µL aliquots of 0.2 mM NaOH; right: speciation diagram of 1 mM 4. The theoretical fit and speciation were carried out with Hyperquad and HySS software[3] using formation constants for Zn(OH) ($10^{-7.84}$) and $Zn(OH)_2$ ($10^{-16.86}$) taken from literature.[4] The fit gives pK_as for the Zn complex 2 of 8.09 and 8.23, and log K_d as 3.94.

Product analysis of the cleavage of BNPP by Zn complexes 3, 4 and 5.

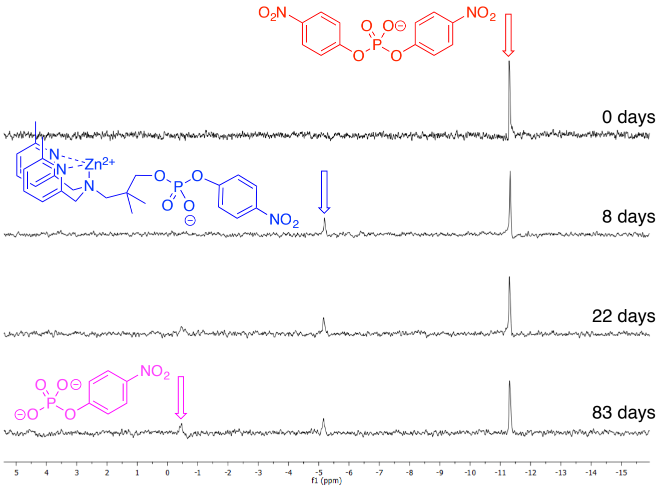

Figure S18. ^{31}**P NMR spectra for the reaction of 5 mM BNPP with 1 mM Zn complex 3 (pH 8.5) at 25 °C. The slow appearance of 4-nitrophenyl phosphate is accounted for by the background reaction of BNPP.**

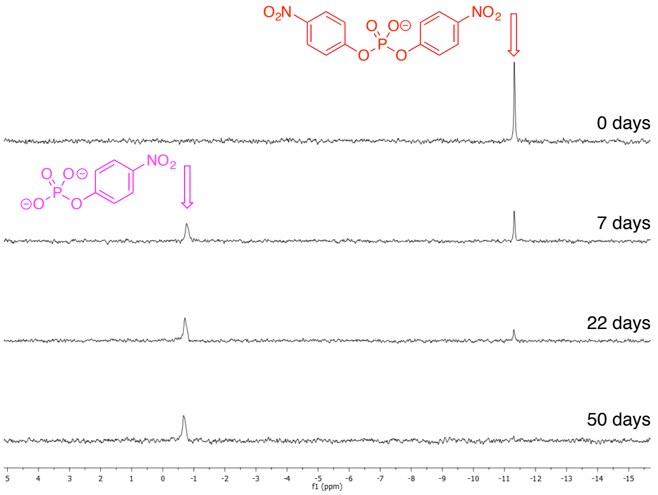

Figure S19. ^{31}**P NMR spectra for the reaction of 5 mM BNPP with 1 mM Zn complex 4 (pH 8.5) at 25 °C.**

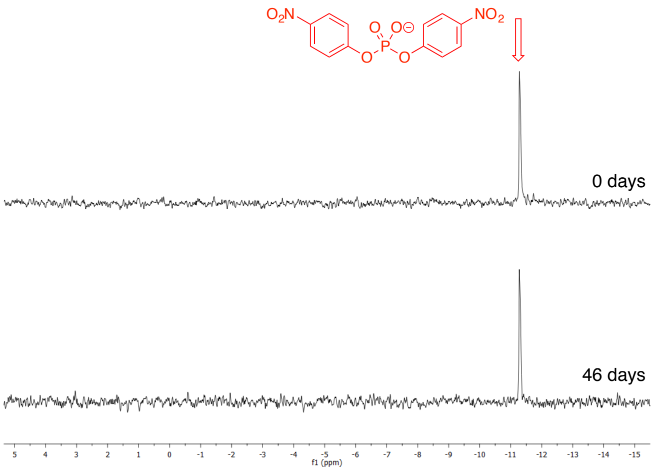

Figure S20. ^{31}**P NMR spectra for the reaction of 5 mM BNPP with 1 mM Zn complex 5 (pH 7.0) at 25 °C.**

Cleavage of BNPP in methanol by Zn complexes 2, 3 and 4'.

The reactions in dry methanol led to the loss of both leaving groups from BNPP, with the second reaction much slower than the first. Thus, a sequential reaction scheme (scheme 2) was used to fit the appearance of both equivalents of 4-nitrophenol (equation S3). This yielded the observed rate constant for the initial reaction between the complex and BNPP, given in tables S3, S4 and S5. Plots of k_{obs} against [complex] show an upward curvature that is very similar to the behaviour of the complexes in water at lower pH. However, in methanol, it can be explained by cooperativity between two complexes as described by Mohamed *et al.*[5] This reaction scheme is given in scheme 3, and leads to equation S4. The k_2 values have been obtained by fitting equation S4 to the data in tables S3, S4 and S5 derived by Mohamed *et al.*[5] Although the active species may be involve a second metal ion complex, the variation in the nucleophile structure is the only change between each complex.

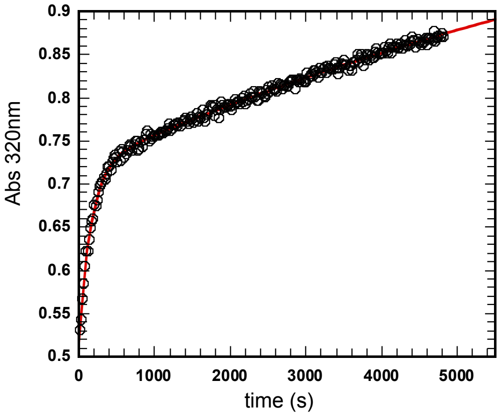

Figure S21. Typical kinetic trace for the cleavage of BNPP (50 µM) in MeOH in the presence of the triflate salt of Zn complex 3 (3mM) at 25 °C (50 mM buffer). The red line is the linear least squares fit of equation S3 to the data.

$$\text{BNPP} \xrightarrow{k_1} \text{I + 4-nitrophenol} \xrightarrow{k_2} \text{P + 4-nitrophenol}$$

Scheme S2.

$$\text{Abs} = \varepsilon_P[A]_0\left(\frac{1}{k_2-k_1}\right)\left\{(2k_2-k_1)\left(1-e^{-k_1t}\right)-k_1\left(1-e^{-k_2t}\right)\right\}+\varepsilon_P[B]_0\left(1-e^{-k_2t}\right)+\varepsilon_P[P]_0$$

Equation S3

$$\text{Complex + BNPP} \underset{K_d}{\rightleftharpoons} \text{Complex.BNPP} \xrightarrow[k]{\text{Complex}} \text{products}$$

Scheme S3.

$$k_{\text{obs}} = k \times \tfrac{1}{2}\{(k_d + [\text{Complex}]_\text{T} + [\text{BNPP}]_\text{T}) - \sqrt{(k_d + [\text{Complex}]_\text{T} + [\text{BNPP}]_\text{T})^2 - 4[\text{Complex}]_\text{T}[\text{BNPP}]_\text{T}\}}$$

Equation S4

[**2**] mM	$_s^s$pH = 8.01	$_s^s$pH = 8.43	$_s^s$pH = 8.81	$_s^s$pH = 9.01	$_s^s$pH = 10.25	$_s^s$pH = 10.73	$_s^s$pH = 11.03
0.5	1.826×10^{-5}	5.882×10^{-5}	2.095×10^{-4}	2.944×10^{-4}	3.759×10^{-4}	3.061×10^{-4}	3.730×10^{-4}
1	7.366×10^{-5}	2.655×10^{-4}	7.836×10^{-4}	1.280×10^{-3}	8.089×10^{-4}	6.680×10^{-4}	6.922×10^{-4}
1.5	1.990×10^{-4}	2.656×10^{-4}	1.537×10^{-3}	2.379×10^{-3}	1.312×10^{-3}	1.033×10^{-3}	1.017×10^{-3}
2	3.140×10^{-4}	9.328×10^{-4}	2.498×10^{-3}	3.519×10^{-3}	2.088×10^{-3}	1.413×10^{-3}	1.424×10^{-3}
2.5	5.104×10^{-4}	1.373×10^{-3}	3.570×10^{-3}	4.936×10^{-3}	2.851×10^{-3}	1.962×10^{-3}	1.778×10^{-3}
3	6.768×10^{-4}	1.822×10^{-3}	4.212×10^{-3}	6.205×10^{-3}	3.798×10^{-3}	2.429×10^{-3}	2.102×10^{-3}

Table S3. Observed rate constants for the cleavage of BNPP (50 µM) catalysed by the triflate salt of Zn complex 2 (50 mM buffer; 25 °C).

[**3**] mM	$_s^s$pH = 8.03	$_s^s$pH = 8.41	$_s^s$pH = 8.61	$_s^s$pH = 8.99	$_s^s$pH = 9.33	$_s^s$pH = 10.33	$_s^s$pH = 10.64
0.5	1.536×10^{-5}	3.225×10^{-5}	1.232×10^{-4}	2.190×10^{-4}	3.161×10^{-4}	2.290×10^{-4}	2.030×10^{-4}
1	9.123×10^{-5}	1.841×10^{-4}	1.301×10^{-3}	1.346×10^{-3}	1.666×10^{-3}	1.170×10^{-3}	9.140×10^{-4}
1.5	1.680×10^{-4}	6.374×10^{-4}	2.005×10^{-3}	2.060×10^{-3}	3.532×10^{-3}	2.240×10^{-3}	1.430×10^{-3}
2	2.860×10^{-4}	7.551×10^{-4}	2.874×10^{-3}	3.580×10^{-3}	5.557×10^{-3}	3.730×10^{-3}	2.360×10^{-3}
2.5	4.080×10^{-4}	1.051×10^{-3}	3.958×10^{-3}	4.970×10^{-3}	7.577×10^{-3}	5.890×10^{-3}	3.260×10^{-3}
3	5.170×10^{-4}	1.470×10^{-3}		6.360×10^{-3}	1.119×10^{-2}	7.270×10^{-3}	4.150×10^{-3}

Table S4. Observed rate constants for the cleavage of BNPP (50 µM) catalysed by the triflate salt of Zn complex 3 (50 mM buffer; 25 °C).

[**4**] mM	$_s^s$pH = 8.05	$_s^s$pH = 8.37	$_s^s$pH = 8.52	$_s^s$pH = 8.89	$_s^s$pH = 9.24	$_s^s$pH = 10.16
0.5	4.130×10^{-6}	6.784×10^{-6}	1.153×10^{-5}	1.344×10^{-5}	2.171×10^{-5}	7.617×10^{-6}
1	1.232×10^{-5}	2.003×10^{-5}	2.562×10^{-5}	3.336×10^{-5}	4.210×10^{-5}	1.956×10^{-5}
1.5	2.021×10^{-5}	3.264×10^{-5}	3.948×10^{-5}	5.321×10^{-5}	7.363×10^{-5}	2.909×10^{-5}
2	2.769×10^{-5}	4.167×10^{-5}	6.065×10^{-5}	8.287×10^{-5}	8.945×10^{-5}	4.117×10^{-5}
2.5	3.741×10^{-5}	6.147×10^{-5}	7.288×10^{-5}	1.035×10^{-4}	1.316×10^{-4}	5.137×10^{-5}
3	4.529×10^{-5}	7.365×10^{-5}	8.813×10^{-5}	1.260×10^{-4}	1.490×10^{-4}	5.880×10^{-5}

Table S5. Observed rate constants for the cleavage of BNPP (50 µM) catalysed by the triflate salt of Zn complex 4' (50 mM buffer; 25 °C).

**2**		**3**		**4'**	
$_s^s$pH	Log k_2 M^{-1} s^{-1}	$_s^s$pH	Log k_2 M^{-1} s^{-1}	$_s^s$pH	Log k_2 M^{-1} s^{-1}
8.01	0.324	8.03	-0.275	8.05	-1.674
8.43	0.423	8.41	0.117	8.37	-1.444
8.81	0.516	8.61	0.478	8.52	-1.450
9.09	0.645	8.99	0.745	8.89	-1.22
10.25	0.437	9.33	1.263	9.24	-1.221
10.73	0.042	10.33	0.994	10.16	-1.635
11.03	-0.150	10.64	0.498		

Table S6. Dependence of the limiting rate constant k_2 for the cleavage of BNPP in dry methanol catalysed by the triflate salts of Zn complexes 2, 3 and 4' on the $_s^spH$ (50 mM buffer; 25 °C).

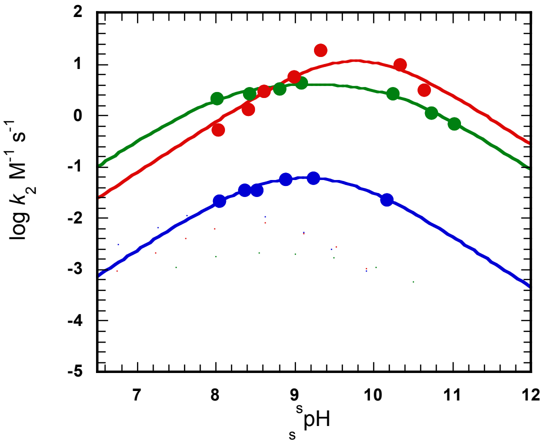

Figure S22. pH rate profile for the cleavage of BNPP in dry methanol (50 mM buffer, 25 °C) catalysed by triflate salts of the Zn complexes 2 (green); 3 (red) and 4' (blue). The solid line is the least squares fit of equation 1 (main text) to the data.

Computational Methods.

Initial geometry optimizations were performed at the Hartree Fock (HF) level of theory using the 6-31+G* basis set. Hay and Wadt's effective core potential with the double-ζ valence basis set (LANL2DZ[6,7]) was used to describe zinc. The resulting structures were characterised by frequency calculations. Single point energy calculations on the optimized structures were performed at the DFT level of theory using the dispersion corrected M06-2X functional[8] with a larger (6-311+G**) basis set, and the metal center described as before. For both optimization and single point calculations, solvation was implicitly accounted for by the SMD continuum solvation model.[9]

All quantum chemical calculations were performed using the Gaussian 09 simulation package.[10] The computational methodology used here is at a similar level to that used by Ohanessian *et al*. for their study of biomimetic Zn complexes.[11] In this study, it was demonstrated that reliable results in terms of geometry and chemical accuracy could be reached by simply performing a HF geometry optimization, followed by a B3LYP energy calculation with a larger basis set. In this work, we used the M06-2X functional (instead of the popular B3LYP) as it also includes dispersion correction.

Computational Results.

Using the crystal structure of **4'** as a starting point, three different Zn-complex structures were generated (Fig. S23). Complex 1-1 is closely related to the crystal structure, with a water molecule replacing the nitrate group coordinated to the Zn ion in the crystal structure, and the methoxy group converted to a hydroxyl. From this optimized structure, two further conformations were generated. By rotating the nucleophile side chain dihedral angles N_1-C_1-C_2-C_3 and C_1-C_2-C_3-O_2, complex 1-2 was generated. Complex 1-3 was obtained by changing the propeller like arrangement[12] of the ligand around the zinc. It was found that all structures are very similar in energy (Table S7). Conformations 1-1 and 1-3 show that the non-coordinated oxygen can readily be positioned close to the free coordination site on the Zn ion, and are almost mirror images as the coordinated water leads to a more symmetrical coordination sphere than when nitrate is coordinated.

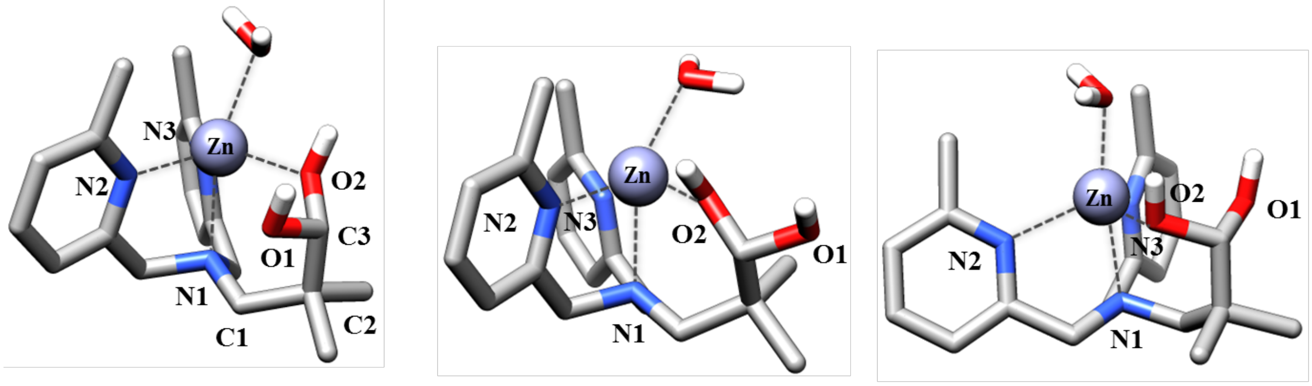

1-1 **1-2** **1-3**

Figure S23: Optimised structures of water-bound zinc-complexes using HF method and 6-31+G* / LANL2DZ basis set.

	Zn-Complex		
**Distance**	**1-1**	**1-2**	**1-3**
**O1-Zn**	3.612	4.424	3.113
**O1-H_2O**	4.591	4.747	3.671
**O2-Zn**	2.274	2.300	2.347
**N1-Zn**	2.349	2.31	2.268
**N2-Zn**	2.285	2.249	2.284
**N3-Zn**	2.258	2.269	2.263
**ΔE**	0.0	-0.40	-0.36

Table S7: Selected distances (in Å) of the optimised structure and relative energy (in kcal mol^{-1}) of the three conformers 1-1, 1-2 and 1-3. The energies were obtained by single point calculation on the optimised structure at M062X and 6-311+G/ LANL2DZ level of theory using SMD solvent model.**

Similar calculations were performed on the monodeprotonated forms (at the non-coordinated hydroxyl) of these complexes. Once again, all the structures show similar energies. In these structures, the deprotonated non-bound oxygen shows a very similar distance to the substrate binding site compared to the protonated forms.

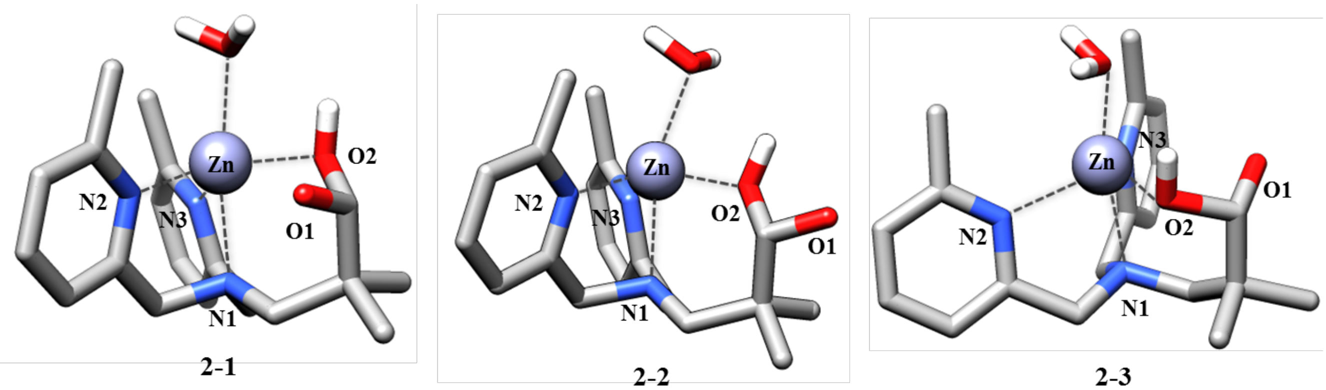

2-1 2-2 2-3

Figure S24: Optimised structure of monodeprotonated water-bound zinc-complexes using HF method and 6-31+G* / LANL2DZ basis set.

	Zn-Complex		
**Distance**	**2-1**	**2-2**	**2-3**
**O1-Zn**	3.559	4.363	3.106
**O1-OH$_2$**	4.664	4.934	3.669
**O2-Zn**	2.200	2.142	2.285
**N1-Zn**	2.296	2.327	2.259
**N2-Zn**	2.294	2.283	2.290
**N3-Zn**	2.327	2.301	2.274
**ΔE**	0.0	-0.02	+0.32

Table S8: Selected distances (in Å) of the optimised structure and relative energy (in kcal mol^{-1}) of the three monodeprotonated conformers 2-1, 2-2 and 2-3. The energies were obtained by single point calculation on the optimised structure using M062X and 6-311+G/ LANL2DZ basis set using SMD solvent model.**

Finally, to explore the possibility of the non-coordinated oxygen acting as a nucleophile towards a coordinated phosphate diester, we replaced the water in the monodeprotonated complexes with methyl-*p*-nitrophenyl-phosphate. As before, we explored the same three different conformations of the complex system. The presence of the phosphate diester reintroduces the asymmetry between 3-1 and 3-3, where it is evident that the non-coordinated oxygen can be either remote or close to the substrate.

3-1 reflects the original crystal structure, and shows a large distance between the non-bound oxygen atom and the phosphate centre (5.60Å). However, once the rotation of the side chain and the propellor inversion have taken place, the non-bound oxygen is much closer to the phosphate centre, reaching a value of 4.06Å with an angle to the leaving group PO bond of 164°.

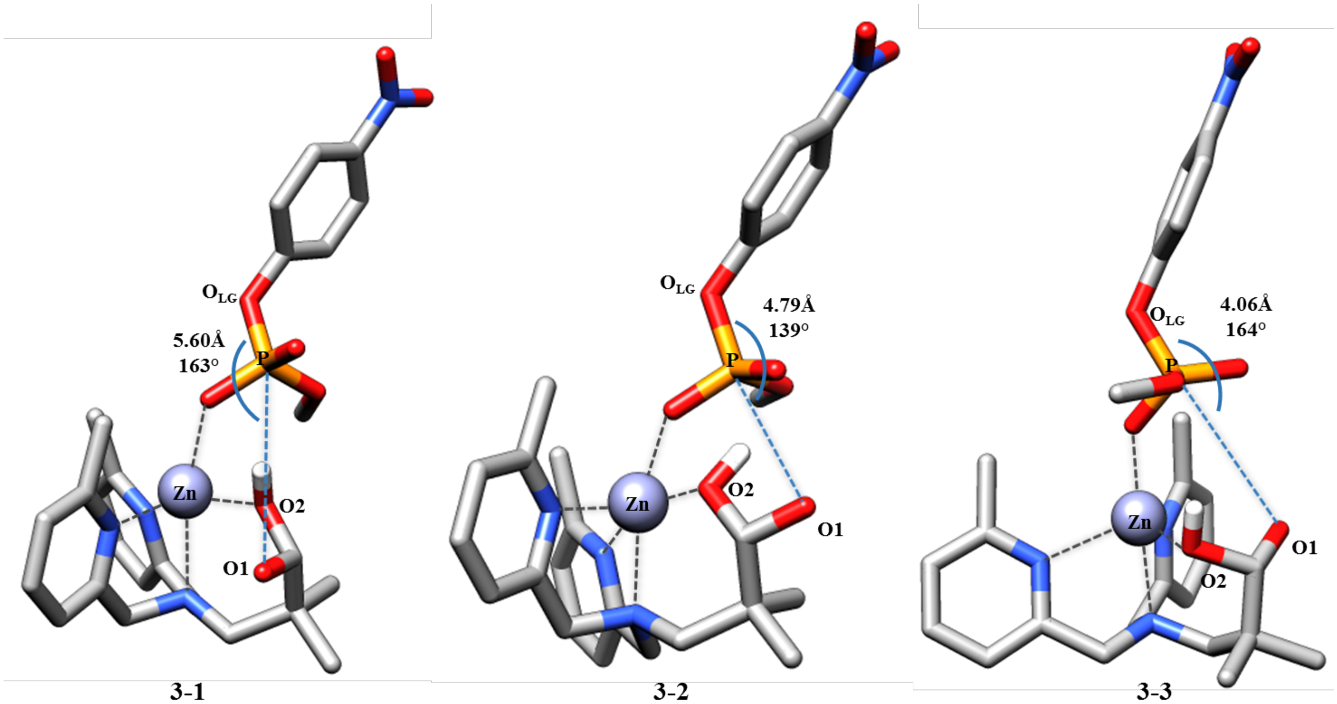

3-1 3-2 3-3

Figure S25 Optimised structure of monodeprotonated zinc complexes with phosphate bound using HF method and 6-31+G* / LANL2DZ basis set.

	Zn-Complex		
**Distance**	**3-1**	**3-2**	**3-3**
**O1-Zn**	2.193	4.359	3.115
**O1-P**	3.436	4.790	4.062
**O1-P-O_{LG}**	165°	139°	164°
**O2-Zn**	3.947	2.157	2.239
**O2-P**	5.604	3.449	3.721
**O2-P-O_{LG}**	163°	137°	154°
**ΔE**	0.0	-0.66	+0.92

Table S9: Selected distances (in Å) and angles (in degrees) of the optimised structures and relative energies (in kcal mol^{-1}) of the three monodeprotonated complexes with the phosphate diester bound. The energies were obtained by single point calculation on the minimised structure using M062X and 6-311+G/ LANL2DZ basis set.**

Finally we performed a transition state optimization for the nucleophile attack reaction of the non-coordinated oxygen for conformation 3-2 (Figure S26). The resulting structure was characterized by frequency calculations, and the minimum energy path connecting reactants to products through this transition state was evaluated by calculating the intrinsic reaction coordinate[13,14] (IRC=ξ) in both the forward and reverse directions in order to identify the corresponding reactant. Our results not only confirm this is a viable pathway for the phosphoryl transfer reaction, but comparison of the reactant stated optimized from the IRC calculation (RS) and the optimized complexes (3-1, 3-2 and 3-3) show almost no difference in energy.

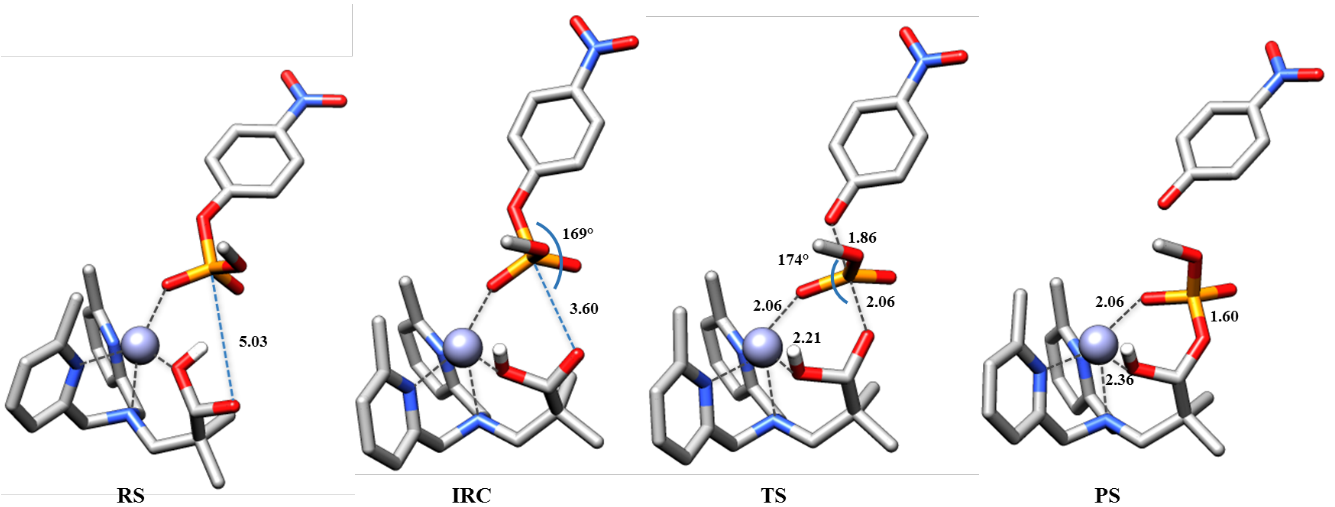

Figure S26: Reactant (RS), last reverse IRC point (IRC), Transition state (TS) and product (PS) state for the phosphoryl transfer reaction. Full optimization of the PS was not possible as the separation between the leaving group and the complex leads to the formation of two independent solvent cavities, so the last point of the IRC is shown here.

	Zn-Complex			
**Distance**	**RS**	**IRC**	**TS**	**PS**
**O1-Zn**	4.336	4.008	3.415	3.582
**O1-P**	5.031	3.600	2.057	1.603
**P-O_{LG}**	1.626	1.628	1.857	3.377
**O1-P-O_{LG}**	160°	169°	174°	172°
**O2-Zn**	2.168	2.183	2.210	2.378
**O2-P**	3.756	3.749	3.431	3.08
**O2-P-O_{LG}**	149°	146°	138°	
**ΔE**	0.0	+2.25	+16.26	

Table S10: Selected distances (in Å) and angles (in degrees) of the structures and relative energies (in kcal mol^{-1}) for the phosphoryl transfer reaction. The energies were obtained by single point calculation on the structure using M062X and 6-311+G/ LANL2DZ basis set.**

Structure: **1-1**

M062X-Energy = -1196.592169/HF-Energy = -1187.789623127/lower frequency = 25.6358 cm^{-1}; Cartesian coordinates:

Zn	11.92721	2.76164	5.43229
N	11.89765	2.25761	3.23117
N	12.32371	0.44839	5.33336
N	10.16083	1.83081	6.54308
O	13.76857	2.75723	6.76658
O	13.09382	1.64552	8.66337
O	12.24100	5.00031	5.64791
C	11.41704	3.05444	2.26922
C	11.44089	2.66868	0.93925
H	11.04954	3.32491	0.18521
C	11.96521	1.42996	0.60701
H	11.99160	1.10890	-0.41839
C	12.43785	0.60591	1.61141
H	12.83301	-0.36911	1.39614
C	12.38598	1.06117	2.91932
C	12.94125	0.19685	4.02922
H	12.83447	-0.84774	3.75023
H	13.99701	0.39790	4.09250
C	10.96232	-0.10731	5.32503
H	10.98643	-1.18361	5.46186
H	10.51536	0.07516	4.35600
C	10.05506	0.51872	6.35731
C	9.10957	-0.24364	7.02104
H	9.05417	-1.30281	6.85420
C	8.24717	0.39593	7.89542
H	7.49904	-0.16390	8.42678
C	8.35982	1.76383	8.08122
H	7.70608	2.28574	8.75388
C	9.33849	2.45890	7.38596
C	13.08446	-0.20771	6.42457
H	13.34959	-1.20903	6.09160
H	12.39409	-0.33094	7.24568
C	14.36434	0.43344	7.01576
C	14.10933	1.77284	7.71470
H	15.01662	2.09536	8.20595
C	10.85625	4.38828	2.67067
H	10.22638	4.29256	3.54790
H	10.26988	4.81271	1.86610
H	11.66042	5.07933	2.90163
C	9.51504	3.94151	7.55149
H	10.49509	4.16026	7.96197
H	9.43175	4.44289	6.59372
C	14.84764	-0.56541	8.08703
H	15.16826	-1.48868	7.61575
H	14.07002	-0.80697	8.80210
H	15.69423	-0.16000	8.63225
C	15.52326	0.62503	6.02399
H	15.33480	1.39492	5.28859
H	15.73694	-0.30381	5.50527
H	16.41987	0.91126	6.56698
H	8.76599	4.34650	8.21990
H	13.88215	3.62272	7.14698
H	12.13521	5.21540	6.57082
H	11.54297	5.46939	5.20093
H	13.12890	2.37925	9.26837

Structure: **1-2**

M062X-Energy = -1196.592808/HF-Energy = -1187.7883587/lower frequency = 33.5338 cm^{-1}; Cartesian coordinates:

Zn	12.06086	2.67945	5.44699
N	11.86405	2.19718	3.23896
N	12.34917	0.39124	5.31957
N	10.19997	1.86570	6.41308
O	14.77224	2.03882	8.88396
H	14.41194	2.42800	9.67446
O	12.96768	2.47163	7.55035
C	11.32393	2.98848	2.30728
C	11.23503	2.58088	0.98476
H	10.79256	3.23026	0.25329
C	11.71490	1.33081	0.63257
H	11.65277	0.99243	-0.38575
C	12.25966	0.51500	1.60915
H	12.62390	-0.46827	1.37780
C	12.31643	0.99072	2.90797
C	12.93497	0.14257	3.99710
H	12.83971	-0.90538	3.72854
H	13.99034	0.36433	4.03213
C	10.98597	-0.16167	5.34904
H	11.00562	-1.22546	5.55883
H	10.53947	-0.04550	4.36964
C	10.07914	0.54447	6.32763
C	9.11710	-0.15322	7.03670
H	9.04929	-1.22136	6.95083
C	8.25292	0.56282	7.84865
H	7.49175	0.05510	8.41277
C	8.38005	1.93941	7.92863
H	7.72530	2.52031	8.55007
C	9.37778	2.56649	7.19616
C	13.16538	-0.24971	6.38155
H	13.57713	-1.16561	5.96896
H	12.48785	-0.55067	7.16947
C	14.31969	0.54167	7.05415
C	13.73963	1.45806	8.14152
H	13.10541	0.88858	8.80683
C	10.82529	4.34170	2.72912
H	10.33961	4.29207	3.69501
H	10.12054	4.73010	2.00475
H	11.65170	5.04259	2.79693
C	9.57612	4.05462	7.25415
H	10.53368	4.29069	7.70648
H	9.56254	4.47904	6.25669
C	15.18905	-0.52001	7.75162
H	15.58630	-1.21431	7.01891
H	14.61332	-1.09153	8.47522
H	16.02550	-0.06828	8.26940
C	15.19445	1.33909	6.07853
H	14.62776	2.07081	5.51445
H	15.68163	0.67167	5.37622
H	15.97344	1.86668	6.61730
H	12.38762	2.86242	8.19599
H	8.79707	4.52405	7.84098
H	13.59839	4.77086	6.02250
O	12.76110	4.77311	5.56582
H	12.94413	5.12077	4.69756

Structure: **1-3**

M062X-Energy = -1196.592735/HF-Energy = -1187.75426644/lower frequency = 39.2026 cm^{-1}; Cartesian coordinates:

Zn	12.18530	2.59708	5.34350
N	14.08651	2.21639	4.17615
N	12.31371	0.33231	5.33154
N	10.34188	1.96903	4.15034
O	13.79212	2.13691	7.96967
O	11.53692	2.09308	7.54231
O	12.00965	4.68418	6.01746
C	15.05477	3.09587	3.90727
C	16.24768	2.69052	3.32711
H	17.01350	3.41256	3.11661
C	16.43064	1.34985	3.03236
H	17.35008	1.01254	2.58943
C	15.41740	0.44874	3.31341
H	15.52280	-0.59770	3.09732
C	14.25111	0.93015	3.88164
C	13.07073	0.01587	4.10925
H	12.41577	0.13054	3.25482
H	13.39989	-1.01747	4.11900
C	10.94095	-0.15919	5.18527
H	10.44728	-0.10428	6.13969
H	10.93370	-1.19966	4.87543
C	10.11441	0.65974	4.21528
C	9.11699	0.05049	3.47251
H	8.96545	-1.01052	3.53819
C	8.33308	0.84104	2.65049
H	7.55010	0.40092	2.06002
C	8.57373	2.20393	2.58920
H	7.98605	2.84135	1.95628
C	9.59692	2.74150	3.35502
C	13.03267	-0.28894	6.47366
H	14.06771	-0.00798	6.34430
H	12.98524	-1.36925	6.35527
C	12.66554	0.01565	7.95162
C	12.58356	1.50754	8.27881
H	12.37096	1.63712	9.33092
C	14.80407	4.53717	4.24702
H	13.89257	4.88327	3.77232
H	15.62483	5.15762	3.91092
H	14.69624	4.66157	5.31838
C	9.90013	4.21287	3.32910
H	9.65555	4.66375	4.28473
H	10.95278	4.38261	3.13338
C	11.37325	-0.66418	8.44317
H	11.35211	-0.65802	9.52902
H	11.34550	-1.69931	8.11892
H	10.46835	-0.17857	8.10702
C	13.81906	-0.59198	8.77588
H	14.78647	-0.21091	8.47293
H	13.83148	-1.67105	8.65901
H	13.68546	-0.37560	9.83122
H	9.32112	4.70851	2.56046
H	13.81115	3.00271	8.36451
H	11.14217	4.81600	6.39090
H	12.05453	5.26566	5.26320
H	11.28340	2.92121	7.93904

Structure: **2-1**

M062X-Energy = -1196.11255/HF-Energy = -1187.3018448/lower frequency = 41.4151 cm^{-1}; Cartesian coordinates:

Zn	12.00264	2.74535	5.47305
N	11.90998	2.26102	3.19890
N	12.32302	0.47722	5.32329
N	10.18978	1.85267	6.55873
O	13.78561	2.80590	6.76002
O	13.11716	1.76631	8.70862
O	12.03279	4.99894	5.52643
C	11.43033	3.04075	2.22277
C	11.45746	2.63473	0.89821
H	11.06738	3.27929	0.13348
C	11.98151	1.39102	0.58454
H	12.00916	1.05452	-0.43594
C	12.45088	0.58286	1.60261
H	12.84481	-0.39640	1.40449
C	12.39721	1.06051	2.90318
C	12.94878	0.21060	4.02601
H	12.84355	-0.83765	3.76034
H	14.00413	0.41247	4.09419
C	10.95971	-0.07408	5.30656
H	10.98152	-1.15098	5.43937
H	10.51645	0.11351	4.33688
C	10.05237	0.54967	6.33957
C	9.08145	-0.20635	6.97444
H	9.00191	-1.25932	6.78006
C	8.22813	0.43118	7.85841
H	7.46132	-0.12276	8.36895
C	8.37647	1.78979	8.08389
H	7.73362	2.30985	8.76846
C	9.37807	2.47759	7.41508
C	13.07277	-0.17555	6.42878
H	13.32725	-1.18206	6.10107
H	12.37291	-0.28550	7.24349
C	14.34512	0.46627	7.03064
C	14.07472	1.79407	7.78219
H	15.03611	2.11621	8.19256
C	10.85828	4.37748	2.59881
H	10.17729	4.28011	3.43635
H	10.32082	4.80813	1.76371
H	11.64944	5.06285	2.88126
C	9.58833	3.94980	7.62720
H	10.59758	4.14013	7.97479
H	9.44446	4.49076	6.69863
C	14.82537	-0.55128	8.08600
H	15.14869	-1.47183	7.60865
H	14.04528	-0.80087	8.79665
H	15.66961	-0.15429	8.64226
C	15.50723	0.65603	6.04223
H	15.32151	1.42072	5.30018
H	15.73478	-0.27286	5.52665
H	16.40052	0.95114	6.58707
H	8.89183	4.33569	8.36065
H	13.72698	3.64297	7.20831
H	12.15932	5.24746	6.43813
H	11.18353	5.35753	5.28625

Structure: **2-2**

M062X-Energy = -1196.112576/HF-Energy = -1187.30067724/lower frequency = 40.7123 cm^{-1}; Cartesian coordinates:

Zn	11.96078	2.76559	5.45559
N	11.87680	2.27416	3.20930
N	12.33581	0.47338	5.31480
N	10.18247	1.82845	6.53875
O	15.02253	2.26937	8.52357
O	13.66985	2.72879	6.74547
C	11.36171	3.04646	2.24715
C	11.37992	2.64788	0.91949
H	10.95916	3.28444	0.16430
C	11.93544	1.42226	0.59228
H	11.95771	1.09156	-0.43022
C	12.44317	0.62099	1.59904
H	12.86124	-0.34541	1.38801
C	12.39357	1.08768	2.90257
C	12.97752	0.24873	4.01823
H	12.92198	-0.79965	3.73761
H	14.02230	0.49954	4.09652
C	10.97848	-0.09039	5.28780
H	11.00575	-1.16816	5.41346
H	10.53510	0.10069	4.31896
C	10.06549	0.52177	6.32443
C	9.10902	-0.24429	6.96826
H	9.04432	-1.29959	6.78127
C	8.24884	0.38651	7.85128
H	7.49334	-0.17667	8.36852
C	8.37307	1.74949	8.06550
H	7.72139	2.26368	8.74611
C	9.36186	2.44857	7.38898
C	13.08479	-0.19163	6.41135
H	13.33917	-1.19308	6.06948
H	12.37773	-0.31557	7.22103
C	14.35526	0.43186	7.04734
C	14.03839	1.75899	7.77939
H	13.12375	1.60474	8.35909
C	10.76532	4.36800	2.64025
H	10.19158	4.27610	3.55400
H	10.11667	4.73853	1.85661
H	11.54847	5.10130	2.80153
C	9.55459	3.92602	7.58318
H	10.53894	4.12674	7.99265
H	9.47175	4.44775	6.63587
C	14.76088	-0.59641	8.12191
H	14.93641	-1.57018	7.67360
H	13.98723	-0.71357	8.87686
H	15.67222	-0.29507	8.62293
C	15.54252	0.59368	6.08786
H	15.40115	1.38760	5.36562
H	15.72682	-0.33017	5.54615
H	16.44185	0.82614	6.64805
H	13.78994	3.59056	7.12912
H	8.81283	4.32602	8.26267
H	12.72950	5.36088	4.81101
O	12.22936	5.05746	5.56308
H	11.37198	5.46320	5.47178

Structure: **2-3**

M062X-Energy = -1196.11203282/HF-Energy = -1187.29514110/ lower frequency = 39.0754 cm^{-1}; Cartesian coordinates:

Zn	12.18370	2.57462	5.36677
N	14.09512	2.21476	4.18841
N	12.31945	0.32049	5.31901
N	10.34080	1.95648	4.15629
O	13.78410	2.20632	8.00318
O	11.53838	2.10650	7.50766
O	11.97198	4.68359	5.99246
C	15.06645	3.09450	3.93181
C	16.26013	2.69378	3.34987
H	17.02808	3.41672	3.15032
C	16.44138	1.35628	3.03997
H	17.36134	1.02210	2.59566
C	15.42576	0.45454	3.30878
H	15.52983	-0.58985	3.08224
C	14.25878	0.93183	3.87997
C	13.07830	0.01508	4.09716
H	12.42520	0.13632	3.24183
H	13.40902	-1.01816	4.09895
C	10.95229	-0.18141	5.16870
H	10.46328	-0.15721	6.12665
H	10.95469	-1.21475	4.83444
C	10.11051	0.64783	4.22061
C	9.09753	0.04346	3.49381
H	8.94404	-1.01730	3.56027
C	8.30246	0.83760	2.68706
H	7.50753	0.40153	2.10967
C	8.54884	2.19957	2.62250
H	7.95449	2.83988	1.99871
C	9.58656	2.73201	3.37195
C	13.03797	-0.29585	6.46530
H	14.07280	-0.01515	6.33338
H	12.99047	-1.37720	6.34619
C	12.67134	0.02770	7.93675
C	12.65508	1.53853	8.26155
H	12.34915	1.63258	9.30756
C	14.81827	4.53237	4.28668
H	13.92399	4.89300	3.79002
H	15.65330	5.15131	3.98453
H	14.67876	4.64094	5.35596
C	9.89606	4.20211	3.33661
H	9.65324	4.66200	4.28853
H	10.94939	4.36630	3.14043
C	11.36729	-0.63049	8.42791
H	11.34237	-0.61754	9.51428
H	11.31633	-1.66957	8.11461
H	10.46983	-0.13191	8.08964
C	13.80706	-0.60924	8.76357
H	14.78329	-0.24561	8.46541
H	13.80272	-1.69005	8.65074
H	13.67624	-0.38934	9.81926
H	9.31920	4.69438	2.56409
H	11.07692	4.80934	6.29567
H	12.06489	5.25810	5.23772
H	11.37790	2.97674	7.85665

Structure: **3-1**

M062X-Energy = -2238.184225/HF-Energy = -2224.93557423/lower frequency = 17.4505 cm^{-1}; Cartesian coordinates:

Zn	11.85964	2.49047	5.99650
N	11.90558	2.57551	3.68411
N	12.31916	0.31565	5.25333
N	9.95391	1.32559	6.43576
O	12.93697	1.92328	7.82100
O	12.42251	0.03812	9.03827
O	12.14311	4.53088	6.49164
C	11.40454	3.54902	2.91627
C	11.59878	3.55329	1.54295
H	11.18232	4.34321	0.94688
C	12.32025	2.52431	0.96135
H	12.48173	2.50636	-0.10127
C	12.80954	1.50783	1.76155
H	13.34760	0.67708	1.34477
C	12.57568	1.57217	3.12573
C	13.10209	0.47631	4.02556
H	13.12755	-0.45394	3.46380
H	14.12184	0.71456	4.28285
C	10.99878	-0.23326	4.90488
H	11.05369	-1.30823	4.76850
H	10.67826	0.18357	3.95930
C	9.92970	0.10242	5.91680
C	8.93397	-0.80776	6.22804
H	8.94666	-1.79232	5.79989
C	7.93454	-0.41413	7.10210
H	7.14538	-1.09447	7.36637
C	7.96477	0.86274	7.63769
H	7.20683	1.19410	8.32190
C	9.00163	1.71450	7.28585
C	12.98779	-0.63179	6.18406
H	13.43716	-1.41946	5.58211
H	12.20743	-1.10031	6.76422
C	14.03892	-0.12648	7.19449
C	13.40237	0.65433	8.37128
H	14.22553	0.94191	9.03246
C	10.61903	4.64741	3.57652
H	10.01684	4.25832	4.38755
H	9.96830	5.12850	2.85699
H	11.28622	5.39982	3.98212
C	9.10242	3.10281	7.85119
H	10.00945	3.20395	8.43780
H	9.13474	3.83754	7.05442
C	14.67201	-1.39664	7.79360
H	15.25259	-1.92532	7.04326
H	13.92386	-2.08125	8.17848
H	15.34152	-1.14273	8.61085
C	15.17539	0.70171	6.57804
H	14.83540	1.62749	6.13249
H	15.70449	0.13358	5.81823
H	15.89672	0.96097	7.34909
H	15.16995	7.84808	6.08729
H	16.19662	9.87310	7.04269
C	14.68320	8.38039	6.88116
C	15.25785	9.51731	7.41608
O	12.87047	6.84189	6.81279

O	16.25362	11.74379	8.56662
C	13.46672	7.93313	7.37681
C	14.59688	10.18291	8.43659
N	15.19611	11.37597	8.99778
P	13.14283	5.31043	7.28241
C	12.81002	8.60744	8.39660
O	14.61867	5.02426	6.77371
C	13.37878	9.74503	8.93492
O	13.15315	5.22600	8.75917
O	14.61318	11.95328	9.87310
H	11.86725	8.24429	8.75833
H	12.88279	10.27526	9.72241
C	14.95213	4.95489	5.38766
H	14.49269	5.76481	4.83891
H	14.63467	4.00659	4.98085
H	16.02600	5.03762	5.32513
H	8.25554	3.32119	8.48928
H	12.53461	2.40836	8.53371

Structure: **3-2**

M062X-Energy = -2238.185274/HF-Energy = -2224.93680032/lower frequency = 21.6397 cm^{-1}; Cartesian coordinates:

Zn	12.08144	2.52978	5.60869
N	12.34182	2.04998	3.31200
N	12.20501	0.27072	5.48555
N	10.00525	1.87711	6.23727
O	13.93941	1.76511	9.47664
O	12.69645	2.38493	7.67142
O	12.77495	4.60704	5.54940
C	12.09007	2.83872	2.26288
C	12.32374	2.40557	0.96583
H	12.11272	3.05637	0.13832
C	12.81516	1.12733	0.76055
H	13.00016	0.76955	-0.23614
C	13.04035	0.30836	1.85213
H	13.39311	-0.69900	1.73280
C	12.78655	0.81310	3.11767
C	13.04589	-0.05675	4.32795
H	12.91080	-1.09711	4.04593
H	14.08142	0.06146	4.60463
C	10.82210	-0.15560	5.21265
H	10.71797	-1.22705	5.34718
H	10.58611	0.05708	4.17785
C	9.80739	0.57446	6.06174
C	8.69654	-0.07462	6.57112
H	8.56793	-1.12996	6.42080
C	7.76584	0.67394	7.27369
H	6.89053	0.20416	7.68433
C	7.97390	2.03231	7.44411
H	7.26861	2.63660	7.98250
C	9.11727	2.60968	6.90942
C	12.69361	-0.44750	6.69517
H	13.04350	-1.42318	6.36772
H	11.82869	-0.63170	7.31895
C	13.77045	0.18548	7.61349
C	13.14349	1.28298	8.51653
H	12.21097	0.87307	8.91460
C	11.54310	4.21562	2.51798
H	10.83782	4.20621	3.33937
H	11.04417	4.59138	1.63327
H	12.34445	4.90165	2.76869
C	9.40298	4.07688	7.05995
H	10.30324	4.22828	7.64517
H	9.55427	4.53374	6.08810
C	14.21157	-0.96530	8.53713
H	14.61158	-1.79190	7.95719
H	13.37813	-1.34553	9.12394
H	14.98161	-0.64101	9.22630
C	15.00315	0.71671	6.87416
H	14.76391	1.51582	6.18157
H	15.49537	-0.07589	6.31776
H	15.72286	1.10658	7.58580
H	13.23473	3.14271	7.89218
H	12.36077	7.62696	8.65120
H	13.17917	9.59507	9.89555
C	13.12130	8.25897	8.23438
C	13.57870	9.36134	8.92942

O	13.15752	6.91448	6.27780
O	14.57969	11.53984	10.16246
C	13.65247	7.97252	6.98476
C	14.55911	10.15473	8.35250
N	15.04093	11.31088	9.07907
P	13.73878	5.40134	6.36479
C	14.62955	8.77418	6.41250
O	15.14682	5.49955	5.63875
C	15.09105	9.87878	7.10253
O	13.99026	5.02759	7.77586
O	15.88359	11.99741	8.57110
H	15.01829	8.53691	5.44104
H	15.84487	10.50693	6.67275
C	15.26832	5.67360	4.22713
H	14.64670	6.48918	3.88516
H	14.99595	4.75948	3.72008
H	16.30499	5.90308	4.03585
H	8.58266	4.58072	7.55528

Structure: **3-3**

M062X-Energy = -2238.18275013/HF-Energy = -2224.93493333/lower frequency = 25.6358 cm^{-1}; Cartesian coordinates:

Zn	12.99939	2.64079	5.85831
N	15.24029	2.31036	5.05926
N	13.31954	0.35352	5.85963
N	11.56106	1.80606	4.20121
O	14.09234	2.30118	8.75574
O	12.00816	2.08986	7.78854
C	16.23289	3.20026	4.99982
C	17.53851	2.80939	4.73275
H	18.31875	3.54548	4.68816
C	17.81131	1.46787	4.53205
H	18.81562	1.14074	4.33204
C	16.77386	0.55270	4.59157
H	16.94341	-0.49660	4.43872
C	15.49751	1.02232	4.85160
C	14.31918	0.08016	4.81888
H	13.85389	0.19347	3.84806
H	14.66768	-0.94581	4.88295
C	12.04060	-0.23024	5.45090
H	11.35074	-0.17322	6.27422
H	12.15817	-1.28123	5.20119
C	11.39634	0.48974	4.28652
C	10.62133	-0.21841	3.38164
H	10.51668	-1.28294	3.47611
C	9.99968	0.47884	2.36125
H	9.39094	-0.03722	1.64098
C	10.18212	1.84866	2.27001
H	9.72392	2.41655	1.48253
C	10.97763	2.48683	3.21020
C	13.81893	-0.22434	7.13186
H	14.83855	0.12105	7.21986
H	13.86423	-1.30656	7.01524
C	13.12847	0.07310	8.48643
C	12.96685	1.58275	8.77456
H	12.43515	1.66787	9.72677
C	15.89627	4.64529	5.22972
H	15.07752	4.95260	4.59024
H	16.75324	5.27381	5.02216
H	15.59780	4.80441	6.25880
C	11.20111	3.97119	3.14605
H	10.69021	4.46609	3.96447
H	12.25655	4.20437	3.21990
C	11.78661	-0.65537	8.69899
H	11.53878	-0.65519	9.75710
H	11.85320	-1.69258	8.38217
H	10.95312	-0.19897	8.18432
C	14.09220	-0.50159	9.54466
H	15.09267	-0.09586	9.45271
H	14.15864	-1.58245	9.45231
H	13.73239	-0.27964	10.54534
H	10.82129	4.37272	2.21515
H	11.65390	2.90572	8.12409
H	9.91096	4.49043	6.90251
H	9.23425	5.42734	8.23944
C	9.95361	5.42912	7.43556
H	9.74349	6.24770	6.76069

O	11.23502	5.59853	8.03986
O	12.56044	4.68968	6.09621
O	12.37994	7.11549	6.36867
P	12.56419	5.71863	7.17902
H	14.41517	8.48812	5.58647
C	12.71116	8.34222	6.86450
C	13.82497	8.98004	6.33595
C	11.92124	8.94976	7.83027
C	14.16363	10.24152	6.78296
H	11.05719	8.44582	8.21824
H	15.02281	10.74315	6.38564
C	13.37479	10.83803	7.75567
C	12.25772	10.21058	8.28491
O	13.70027	5.83142	8.11708
O	14.67743	12.70785	7.74421
N	13.72903	12.15816	8.23202
H	11.65868	10.69105	9.03175
O	13.06249	12.65451	9.09750

Structure: **RS**

M062X-Energy = -2238.18312031//HF-Energy = -2224.93484844/lower frequency =18.9483; Cartesian coordinates:

Zn	-1.07084600	-1.10856400	0.04378400
N	-1.27144500	-1.05608200	-2.22383200
N	-1.26753700	-3.28665100	-0.57865600
N	-3.24164600	-1.67226500	0.64960400
O	0.96721600	-2.91259400	3.41950500
O	-0.44112000	-1.84120700	1.98419600
O	-0.22793400	0.77723000	0.48086100
C	-1.56441800	-0.00401700	-2.99570200
C	-1.47864300	-0.08174700	-4.37764500
H	-1.72347000	0.77467600	-4.97711700
C	-1.08240400	-1.27262700	-4.96333000
H	-1.00917500	-1.35488000	-6.03260800
C	-0.80046600	-2.35995500	-4.15640500
H	-0.51140100	-3.30696700	-4.57242900
C	-0.90762300	-2.20636600	-2.78414300
C	-0.58412700	-3.37080600	-1.87349300
H	-0.83921100	-4.29712100	-2.38076200
H	0.48192200	-3.38274200	-1.71071700
C	-2.70359600	-3.53863700	-0.78903400
H	-2.90070900	-4.60415700	-0.83564500
H	-2.99824700	-3.12676500	-1.74503900
C	-3.58362200	-2.89247400	0.25304800
C	-4.73397300	-3.51905500	0.70217000
H	-4.97714500	-4.51043000	0.36874200
C	-5.55313900	-2.83113600	1.58196200
H	-6.45601300	-3.28389300	1.94993100
C	-5.19659800	-1.55478700	1.98412600
H	-5.81280700	-0.99731300	2.66387700
C	-4.01967700	-1.00099700	1.49946600
C	-0.74106000	-4.31104200	0.36401000
H	-0.51662600	-5.19932900	-0.22096600
H	-1.55927600	-4.57905100	1.01979500
C	0.47041700	-3.99972200	1.27779800
C	0.03400700	-3.13034800	2.48790200
H	-0.86635700	-3.59317500	2.90179800
C	-1.98295100	1.27663200	-2.32950300
H	-2.60552600	1.08105600	-1.46596400
H	-2.53369300	1.89929000	-3.02336900
H	-1.11157300	1.83141800	-1.99860600
C	-3.57253900	0.37283100	1.91339400
H	-4.32393700	0.85318800	2.52752100
H	-2.65157000	0.31217900	2.48308000
C	0.89110200	-5.36880300	1.84290400
H	1.17067400	-6.04644300	1.04162000
H	0.08188500	-5.83355900	2.40209600
H	1.74090100	-5.27214700	2.50760900
C	1.66893100	-3.38581500	0.54755800
H	1.44261500	-2.42332500	0.10350200
H	2.02132800	-4.04529100	-0.24027100
H	2.49240900	-3.24101200	1.23838400
H	0.08644100	-1.18297300	2.42290300
H	0.79694800	4.75085600	-1.42595100
H	2.48726100	6.46877700	-1.94285300
C	1.76453900	4.72320500	-0.96229100
C	2.71050900	5.68285800	-1.25001300

O	1.06398000	2.82235900	0.18637900
O	4.68783600	7.45639100	-1.73632200
C	2.06214100	3.70349100	-0.06265300
C	3.94943000	5.60927100	-0.62713900
N	4.94706100	6.61040200	-0.92479900
P	1.17888200	1.25866500	0.61554900
C	3.30317400	3.64015100	0.56000700
O	2.27062600	0.60032400	-0.13256500
C	4.25438500	4.60043500	0.27243000
O	1.59430600	1.31108300	2.14472600
O	6.00058600	6.56118100	-0.35145400
H	3.53291100	2.86352700	1.26133500
H	5.21367300	4.55918900	0.74738400
H	-3.38814600	0.99231500	1.04302700
C	0.73472000	1.85503800	3.14673700
H	1.27020400	1.78384300	4.08019900
H	0.51601900	2.89182000	2.92964000
H	-0.18168300	1.28621300	3.20404000

Structure: **TS**

M062X-Energy = -2238.15721268/HF-Energy= -2224.88307557/lower frequency = -355.9064; Cartesian coordinates:

Zn	12.06251300	2.31927000	5.57481900
N	11.91689000	2.10216000	3.36802800
N	12.08176900	0.05285900	5.24247900
N	10.00591700	1.72031400	6.30779300
O	14.77235400	2.23984600	7.71099100
O	12.55659900	1.60330900	7.60687300
O	13.48678600	3.74251100	6.01486500
C	11.57659400	3.09660200	2.54116000
C	11.38827400	2.86809900	1.18808000
H	11.11154300	3.67784400	0.53987900
C	11.55636500	1.58312200	0.69504400
H	11.40837900	1.38072800	-0.35029200
C	11.90829900	0.56363700	1.56130700
H	12.04051600	-0.44432600	1.21528300
C	12.08630800	0.87004200	2.90126900
C	12.54617500	-0.19382500	3.87397900
H	12.22000200	-1.16878800	3.52424700
H	13.62207400	-0.20134400	3.85290400
C	10.67751400	-0.38830700	5.33130000
H	10.62387200	-1.44548100	5.56763800
H	10.21780100	-0.26274000	4.35841300
C	9.83223700	0.40332300	6.30138200
C	8.86697100	-0.21134300	7.07931500
H	8.75176900	-1.27870100	7.05998800
C	8.06184400	0.58621600	7.87790600
H	7.30253500	0.14274400	8.49630600
C	8.24887800	1.95799100	7.87819000
H	7.64436500	2.59985500	8.49073300
C	9.24635800	2.49777300	7.07748500
C	12.90691300	-0.66433800	6.25041400
H	13.17184300	-1.63466400	5.83552200
H	12.26849500	-0.85703500	7.10064600
C	14.20187800	0.00729700	6.77805700
C	13.91748000	1.19211300	7.77481400
H	13.95559900	0.77482100	8.77908000
C	11.42259700	4.46840600	3.13490700
H	10.80234600	4.43357900	4.02339200
H	10.97388800	5.14881700	2.42253400
H	12.39231200	4.86302900	3.42006100
C	9.51466400	3.97603500	7.03956600
H	8.90626200	4.49875500	7.76669900
H	10.55924500	4.17862500	7.25144000
C	14.91677500	-1.08546100	7.59803800
H	15.25237300	-1.89727500	6.95986200
H	14.26436900	-1.50487100	8.35944600
H	15.78866800	-0.67456100	8.09913100
C	15.16273500	0.41951100	5.65540900
H	14.78926000	1.23365000	5.04799000
H	15.36468700	-0.42785800	5.00686700
H	16.10896100	0.73594100	6.07464100
H	12.39003000	2.31393000	8.21685500
H	14.64289300	7.78270600	4.46441500
H	16.26474200	9.61456900	4.39192300
C	15.51734800	7.75516000	5.08795100
C	16.42598400	8.78116400	5.04632900

O	14.79561100	5.70555600	5.90282700
O	18.33682400	10.69717700	5.05015200
C	15.70757900	6.64054600	5.93478600
C	17.55728700	8.72525000	5.86099700
N	18.51393600	9.78149600	5.81455400
P	14.81013600	4.01543000	6.67234100
C	16.85257900	6.61554800	6.75613500
O	16.16417500	3.65374100	6.17680500
C	17.76422900	7.64519000	6.71418700
O	14.79141500	4.67469800	8.12558200
O	19.47723800	9.73283300	6.53857600
H	17.02912100	5.79199900	7.41497200
H	18.63313800	7.60681300	7.34081100
H	9.29035500	4.37358300	6.05514200
C	13.61441500	5.22865400	8.69288800
H	13.92256700	5.74493800	9.58977500
H	13.14918000	5.93042800	8.01494000
H	12.91411700	4.44637200	8.95373300

References

(1) Feig, A. L.; Becker, M.; Schindler, S.; van Eldik, R.; Lippard, S. J. *Inorg. Chem.* **1996**, *35*, 2590–2601.

(2) Isoda, N.; Torii, Y.; Okada, T.; Misoo, M.; Yokoyama, H.; Ikeda, N.; Nojiri, M.; Suzuki, S.; Yamaguchi, K. *Dalton Trans*. **2009**, 10175–7.

(3) Gans, P.; Sabatini, A.; Vacca, A. *Talanta*, **1996**, *43*, 1739-1753.

(4) Reichle, R.; McCurdy, K.; Helper, L. *Can. J. Chem*. **1975**, *53*, 3841–3845.

(5) Mohamed, M. F.; Sánchez-Lombardo, I.; Neverov, A. A.; Brown, R. S. *Org. Biomol. Chem*. **2012**, *10*, 631–9.

(6) Hay, P. J.; Wadt, W. R. *J. Chem. Phys.* **1985**, *82*, 270.

(7) Wadt, W. R.; Hay, P. J. *J. Chem. Phys.* **1985**, *82*, 284.

(8) Zhao, Y.; Truhlar, D. G. *Theor. Chem. Acc*. **2008**, *120*, 215.

(9) Marenich, A. V.; Cramer, C. J.; Truhlar, D. G. *J. Phys. Chem. B* **2009**, *113*, 6378.

(10) Frisch, M. J.; Trucks, G. W.; Schlegel, H. B.; Scuseria, G. E.; Robb, M. A.; Cheeseman, J. R.; Scalmani, G.; Barone, V.; Mennucci, B.; Petersson, G. A.; Nakatsuji, H.; Caricato, M.; Li, X.; Hratchian, H. P.; Izmaylov, A. F.; Bloino, J.; Zheng, G.; Sonnenberg, J. L.; Hada, M.; Ehara, M.; Toyota, K.; Fukuda, R.; Hasegawa, J.; Ishida, M.; Nakajima, T.; Honda, Y.; Kitao, O.; Nakai, H.; Vreven, T.; Montgomery Jr., J. A.; Peralta, J. E.; Ogliaro, F.; Bearpark, M.; Heyd, J. J.; Brothers, E.; Kudin, K. N.; Staroverov, V. N.; Kobayashi, R.; Normand, J.; Raghavachari, K.; Rendell, A.; Burant, J. C.; Iyengar, S. S.; Tomasi, J.; Cossi, M.; Rega, N.; Millam, J. M.; Klene, M.; Knox, J. E.; Cross, J. B.; Bakken, V.; Adamo, C.; Jaramillo, J.; Gomperts, R.; Stratmann, R. E.; Yazyev, O.; Austin, A. J.; Cammi, R.; Pomelli, C.; Ochterski, J. W.; Martin, R. L.; Morokuma, K.; Zakrzewski, V. G.; Voth, G. A.; Salvador, P.; Dannenberg, J. J.; Dapprich, S.; Daniels, A. D.; Farkas, Ö.; Foresman, J. B.; Ortiz, J. V.; Cioslowski, J.; Fox, D. J. Gaussian 09, Revision C01; Gaussian, Inc.: Wallingford CT, 2009.

(11) Frison, G.; Ohanessian, G. *J. Comput. Chem*. **2008**, *29*, 416.

(12) Canary, J. W.; Allen, C. S.; Castagnetto, J. M.; Chiu, Y. H.; Toscano, P. J.; Wang, Y. H. *Inorg. Chem*. **1998**, *37*, 6255.

(13) van der Ploeg, J. R.; Eichhorn, E.; Leisinger, T. *Arch. Microbiol*. **2001**, *176*, 1.

(14) Hratchian, H. P.; Schlegel, H. B. *J. Chem. Theory Comput*. **2005**, *1*, 61.
